# GREB1 isoform 4 is specifically transcribed by MITF and required for melanoma proliferation

**DOI:** 10.1038/s41388-023-02803-6

**Published:** 2023-09-01

**Authors:** Koei Shinzawa, Shinji Matsumoto, Ryota Sada, Akikazu Harada, Kaori Saitoh, Keiko Kato, Satsuki Ikeda, Akiyoshi Hirayama, Kazunori Yokoi, Atsushi Tanemura, Keisuke Nimura, Masahito Ikawa, Tomoyoshi Soga, Akira Kikuchi

**Affiliations:** 1 Department of Molecular Biology and Biochemistry, Graduate School of Medicine, Osaka University, Suita, Osaka Japan; 2 Integrated Frontier Research for Medical Science Division, Institute for Open and Transdisciplinary Research Initiatives (OTRI), Osaka University, Suita, Osaka Japan; 3 Institute for Advanced Biosciences, Keio University, Tsuruoka, Yamagata Japan; 4 Department of Dermatology, Graduate School of Medicine, Osaka University, Suita, Osaka Japan; 5 Department of Genome Biology, Graduate School of Medicine, Osaka University, Suita, Osaka Japan; 6 Gunma University Initiative for Advanced Research, Gunma University, Maebashi, Gunma Japan; 7 Research Institute for Microbial Diseases, Osaka University, Suita, Osaka Japan; 8 Center for Infectious Disease Education and Research, Osaka University, Suita, Osaka Japan

**Keywords:** Melanoma, Cancer genetics, Transcription, DNA metabolism

## Abstract

Growth regulation by estrogen in breast cancer 1 (GREB1) is involved in hormone-dependent and -independent tumor development (e.g., hepatoblastoma). In this study, we found that a GREB1 splicing variant, isoform 4 (Is4), which encodes C-terminal half of full-length GREB1, is specifically expressed via microphthalmia-associated transcription factor (MITF) in melanocytic melanoma, and that two MITF-binding E-box CANNTG motifs at the 5’-upstream region of *GREB1* exon 19 are necessary for *GREB1 Is4* transcription. MITF and GREB1 Is4 were strongly co-expressed in approximately 20% of the melanoma specimens evaluated (17/89 cases) and their expression was associated with tumor thickness. GREB1 Is4 silencing reduced melanoma cell proliferation in association with altered expression of cell proliferation-related genes in vitro. In addition, GREB1 Is4 targeting by antisense oligonucleotide (ASO) decreased melanoma xenograft tumor formation and GREB1 Is4 expression in a *BRAF*^*V600E*^*; PTEN*^*flox*^ melanoma mouse model promoted melanoma formation, demonstrating the crucial role of GREB1 Is4 for melanoma proliferation in vivo. GREB1 Is4 bound to CAD, the rate-limiting enzyme of pyrimidine metabolism, and metabolic flux analysis revealed that GREBI Is4 is necessary for pyrimidine synthesis. These results suggest that MITF-dependent GREB1 Is4 expression leads to melanoma proliferation and GREB1 Is4 represents a new molecular target in melanoma.

## Introduction

Melanoma is a skin cancer that originates from melanocytes and causes 80% of skin cancer-related deaths [1]. Melanoma development involves a complex interaction between environmental factors and genetic alterations. Some melanomas arise from pre-existing nevi, benign lesions formed from melanocytes harboring oncogenic mutations [2]. However, a single gene mutation cannot drive their transformation into fully malignant tumors, and many pathogenic alterations are required [3]. The most common types of melanoma in Caucasians are found on sun-exposed skin. Somatic mutations in *BRAF*, *NRAS*, or *neurofibromatosis 1 (NF1)* are frequently observed in melanoma and their frequencies have been shown to depend on the cumulative amount of UV light and the anatomical site. Acral melanoma is another form of melanoma common in East Eurasian with a higher degree of chromosomal aberrations, including an increased *cyclin D1 (CCND1)* copy number [4].

Understanding the molecular pathogenesis of melanoma has led to the development of new anti-cancer drugs [5]. Molecular targeted therapy with BRAF inhibitor has been approved to treat *BRAF*^*V600E*^-mutated melanoma [6]. Recently, antibodies against CTLA-4 and PD-1, which inhibit the immune checkpoints, have shown remarkable therapeutic effects. However, in acral and mucosal melanoma, single nucleotide mutations causing amino acid changes are extremely rare, making anti-PD-1 antibodies less effective than in the Caucasian population [7]. Therefore, developing the treatment of melanoma where immune checkpoint inhibitors do not work is needed.

The microphthalmia-associated transcription factor (MITF) pathway may also be involved in melanoma cell proliferation [8]. MITF is a basic helix-loop-helix leucine zipper (bHLH-LZ) transcription factor that binds DNA as a dimer [9]. There are ten MITF isoforms; MITF-M (here designated MITF) is the most abundant isoform in melanocytes and plays a key role in melanocyte differentiation [10]. In addition, several MITF target genes are involved in the growth and survival of melanocytes. MITF is frequently amplified in melanoma, and its ectopic expression with BRAF^V600E^ transforms normal melanocytes into immortalized melanocytes [11]. Thus, unidentified MITF target genes may represent new molecular targets for melanoma therapy.

*Growth regulation by estrogen in breast cancer 1 (GREB1)* is a gene induced by estrogen in MCF7 breast cancer cells [12]. It is expressed in estrogen receptor α (ERα)-positive breast cancer cells but not ERα-negative cells. ERα binds to the *GREB1* promoter and promotes GREB1 expression, which in turn forms a complex with ERα and activates transcriptional activity of ERα [13, 14]. Although GREB1 has been shown to be involved in the formation of hormone-dependent tumors, GREB1 was also found to be expressed in hepatoblastomas and hepatocellular carcinoma, which is a hormone-independent malignant tumor, and to promote cell proliferation [15, 16].

Nucleotide synthesis is a key event in cell proliferation. The enzymes involved in this process are attractive therapeutic targets in cancer cells [17]. Nucleotide biosynthesis utilizes ribose 5-phosphate produced from the pentose phosphate pathways and nonessential amino acids [18]. The multifunctional enzyme CAD catalyzes the rate-limiting step in the pyrimidine biosynthesis pathway [19, 20]. Indeed, inhibition of the dihydroorotate dehydrogenase (DHODH) enzyme downstream of CAD suppressed growth of glioblastoma, triple negative breast cancer, and colon cancer, demonstrating the importance of pyrimidine synthesis in tumor growth [21–23].

Here, we show that the expression of the GREB1 splicing variant, isoform 4 (Is4), is mediated by MITF in melanoma cells. GREB1 Is4 was highly expressed with MITF in both benign nevi and melanoma samples. Both in vitro and in vivo, the GREB1 Is4 expression stimulated melanoma cell proliferation. GREB1 Is4 bound to and activated CAD, regulating the pyrimidine metabolic pathway. These results suggest that GREB1 Is4, whose expression is induced by MITF, is involved in melanoma cell proliferation via pyrimidine synthesis regulation and that GREB1 Is4 represents a new molecular target for melanoma therapy.

## Results

### GREB1 Is4 is expressed in melanoma cells

**Fig. 1:**
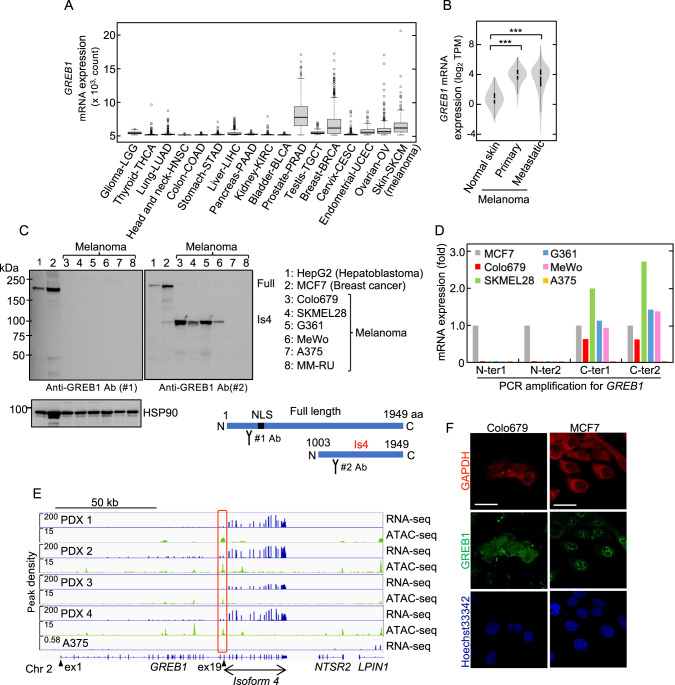
GREB1 Is4 is expressed in melanoma cells. **A** *GREB1* mRNA expression in 17 cancer types was obtained from the TCGA database. The normal distribution across the dataset is visualized with box plots, showing the median and 25th and 75th percentiles. Points are displayed as outliers if they were above or below 1.5 times the interquartile range. The abbreviations used for all cancers are shown in https://gdc.cancer.gov/resources-tcga-users/tcga-code-tables/tcga-study-abbreviations. **B** *GREB1* mRNA expression in normal skin (*n* = 556), primary sites (*N* = 102), or metastatic cutaneous melanoma (*N* = 367) from the GTEx and the TCGA datasets. The distribution of the data is visualized with violin plot. Black boxes span the first quartile to the third quartile; white lines in the black boxes indicate the median; the width of a violin plot indicates the kernel density of the expression values (***P < 0.001 by the Student’s *t* test). **C** Lysates from various cancer cells were probed with the indicated antibodies. Full or Is4 refer to full-length GREB1 or GREB1 Is4, respectively. GREB1 and GREB1 Is4 protein structure and antibody recognition sites were illustrated. Specificity of anti-GREB1antibodies (#1) and (#2) was checked in Supplementary Fig. S1B. HSP90 was used as a loading control. **D** Melanoma-specific *GREB1 Is4* mRNA transcription in various cancer cells was examined by qPCR using region-specific primers. The amplified regions are depicted in Supplementary Fig. S1D. The data are presented as the fold-change relative to the *GREB1* mRNA levels detected in MCF7 cells. **E** Transcribed regions and open chromatin loci of the *GREB1* gene locus in PDX established from four melanoma tissues from three patients were plotted. PDX1 and 2 were established from the same patient. The peak levels of human *GREB1* mRNA transcription and chromatin accessibility were obtained from the RNA- and ATAC-seq data, respectively. The RNA-seq data of A375 cells derived from GEO datasets (GSM1138787) was plotted as a negative control. The closed triangles or red box show exon 1 and 19 or putative *GREB1 Is4* promoter, respectively. Exon numbers were assigned based on the GREB1 transcription ID: ENST00000381486.7 (GREB1-204). **F** Colo679 and MCF7 cells were stained with anti-GREB1 (#2) or anti-GAPDH antibody and counterstained with Hoechst33342. GAPDH or Hoechst33342 was used as cytosolic or nuclear marker, respectively. Scale bars in **F**, 20 μm.

The TCGA dataset showed that in addition to hormone-dependent tumors, such as prostate (PRAD), breast (BRCA), endometrial (UCEC), or ovarian (OV) cancers, *GREB1* mRNA is highly expressed in skin (SKCM, melanoma) cancer (Fig. 1A). The GENT2 dataset also showed high GREB1 expression in skin cancer cell lines (Supplementary Fig. S1A). The human melanoma patient TCGA and GTEx dataset revealed that *GREB1* mRNA expression in primary and metastatic melanoma is higher than in normal skin tissue (Fig. 1B). When GREB1 protein expression was examined in cancer cell lines using an anti-GREB1 antibody (#1) that recognizes the N-terminal region of GREB1 (Supplementary Fig. S1B), full-length GREB1 (216 kDa) was detected only in HepG2 (hepatoblastoma) and MCF7 (breast cancer) but not in melanoma cell lines (Fig. 1C, left blot). When another anti-GREB1 antibody (#2) that recognizes C-terminal half of GREB1, especially fragment A (amino acid Met1003 to Val1270) (Supplementary Fig. S1B), was used, a band at 100 kDa was detected in Colo679, SKMEL28, G361, and MeWo cells, but not in A375 or MM-RU cells (Fig. 1C, right blot). It has been reported that there are mainly three types of melanoma, including melanocytic (proliferative), mesenchymal-like (invasive), and neural crest stem cell (NCSC)-like, depending on MITF-regulated transcriptional states [24]. The Heuristic Online Phenotype Prediction (HOPP) classified SKMEL28, G361, and MeWo cells, which expressed GREB1 Is4, as melanocytic melanoma (Supplementary Fig. S1C) [25].

Overall *GREB1* mRNA transcription was examined in melanoma cell lines by quantitative PCR (qPCR) using the four specific primer sets (Supplementary Fig. S1D). Although all regions of GREB1 were amplified in MCF7 cells, only the C-ter1 and C-ter2 regions were amplified in melanoma cell lines except for A375 cells (Fig. 1D). RNA-seq from patient-derived xenografts (PDXs) of Japanese melanoma cases showed that *GREB1* mRNA transcription starts at exon 19 and matched the GREB1 Is4, which encodes C-terminal half (amino acids 1003–1949) (Fig. 1E). RNA-seq of public GEO showed no expression of *GREB1* in A375 cells (Fig. 1E). ATAC-seq of four Japanese melanoma PDXs revealed extensive chromatin accessibility around the 5’-upstream region of exon 19, where the transcription of *GREB1 Is4* starts (Fig. 1E) (UniProtKB, Q4ZG55-4; Ensembl, GREB1-206). In addition, TCGA dataset showed that *GREB1 Is4* mRNA transcription from exon 19 was elevated only in Skin-SKCM and Uveal-UVM melanomas among 11 cancers (Supplementary Fig. S1E). GREB1 transcripts of Breast-BRCA and Prostate-PRAD primarily encoded full-length GREB1, but GREB1 mRNA transcription levels were low in other cancers (Supplementary Fig. S1E). Taken together, these results indicate that *GREB1 Is4* mRNA is uniquely transcribed in melanoma.

Analysis of publicly available GTEx data demonstrated that full-length GREB1 is transcribed in normal cervix and ovary tissues, while transcription from exon 19 (Is4) predominates in normal skin, albeit at a relatively low expression level (Supplementary Fig. S1F). Furthermore, the FANTOM5 SSTAR (Semantic catalog of Samples, Transcription initiation And Regulators) data revealed that transcription initiation of GREB1 Is4, denoted as p2@GREB1, primarily occurs in melanoma and melanocytes among various human primary cell types, cancer cell lines, and tissues. This information can be accessed at the following link: (https://fantom.gsc.riken.jp/5/sstar/FFCP_PHASE2:Hg19::chr2:11752415..11752445,%2B). Taken together, GREB1 Is4 is expressed in melanocytes.

GREB1 is localized to the nucleus. Because GREB1 Is4 lacks the nuclear localization sequence (NLS) (amino acids 310–319), GREB1 Is4 was mainly localized to the cytoplasm in Colo679 cells, whereas full-length GREB1 was observed as nuclear foci in MCF7 cells (Fig. 1F). Thus, GREB1 Is4 may have unknown functions in the cytosol of melanoma cells.

### MITF stimulates *GREB1 Is4* gene expression

**Fig. 2:**
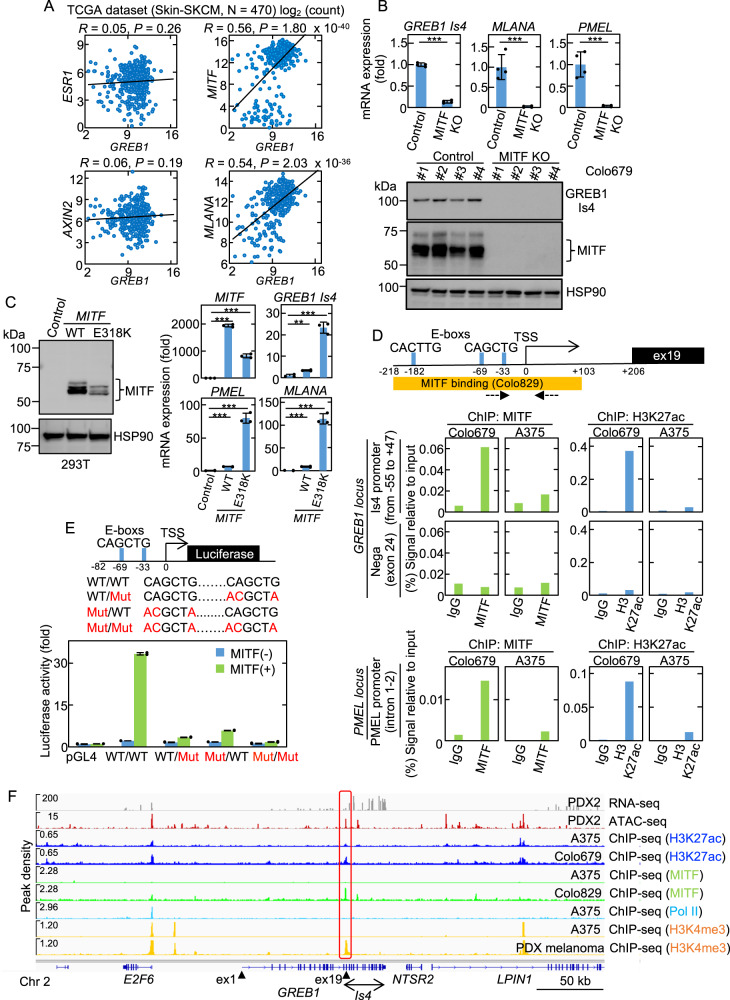
MITF stimulates *GREB1 Is4* gene expression. **A** Scatter plots between the *GREB1* gene (x-axis) and *ESR1*, *AXIN2*, *MITF*, or *MLANA* (y-axis) from TCGA Skin-SKCM (*N* = 470). The solid line indicates a linear fit. *R* and *P* represent the Pearson’s correlation coefficient and *P*-value, respectively. **B** *GREB1 Is4*, *MLANA* and *PMEL* mRNA expression in control and MITF KO Colo679 clones. The qPCR data are shown as the fold to control and presented as the mean ± s.d. from four independent clones (***P < 0.001, Student’s *t* test). Lysates from control vector and MITF KO Colo679 clones were probed with the indicated antibodies. HSP90 was used as a loading control. **C** Left panel, 293T cell lysate expressing WT MITF or MITF^E318K^ was probed with the indicated antibodies. Right panels, *MITF*, *GREB1 Is4*, *PMEL*, and *MLANA* mRNA expression in 293T cells expressing WT MITF or MITF^E318K^. The qPCR data are shown as the fold to control and presented as the mean ± s.d. from three independent experiments (**P < 0.01; ***P < 0.001, Student’s *t* test). **D** Top panel, schematic representation of the putative *GREB1 Is4* promoter region. TSS and MITF binding sites were predicted as described in Materials and Methods. The dashed arrows indicate the primer set of ChIP. Bottom panels, ChIP qPCR by anti-MITF or H3K27ac antibody of Is4 promoter (from −55 to +47) or control region (exon 24) at *GREB1 locus*, or PMEL promoter (Intron 1-2) at *PMEL locus* in Colo679 and A375 cells. The signals were presented as percentage of inputs. **E**. Top panel, schematic representation of the *GREB1 Is4* promoter constructs for transcriptional reporter assay. The WT and Mut constructs of MITF-binding E-box CANNTG motif are shown. Bottom panels, WT/WT, WT/Mut, Mut/WT, or Mut/Mut GREB1 Is4 promoter (-82-0) activity without or with MITF. The luciferase activities are shown as the fold-change relative to the pGL4 vector control and presented as the mean ± s.d. from two independent experiments. F. RNA- and ATAC-seq from Japanese PDX, and ChIP-seq of H3K27ac, MITF, Pol II, and H3K4me3 from public ChIP data in the putative *GREB1 Is4* promoter. The closed triangles or red box show exons 1 and 19 or putative *GREB1 Is4* promoter, respectively.

GREB1 expression is regulated by ERα in breast cancer and by TCF4 in hepatoblastoma [13, 15]. Positive correlation between the mRNA expression of *GREB1* and *ESR1* or *AXIN2*, which are target genes for ERα or TCF4, respectively, was also observed in these patients (Supplementary Fig. S2A). However, in melanoma, there was no correlation between the mRNA expression of *GREB1* and *ESR1* or *AXIN2*, but a strong positive correlation of mRNA expression of *GREB1* with that of *MITF* or *MLANA*, one of MITF target genes [26, 27] in TCGA melanoma and cell lines data (Fig. 2A and Supplementary Fig. S2B). Furthermore, among the top 25 genes highly correlated with *GREB1* gene expression in melanoma, 16 genes were known targets of MITF, such as *MLANA* and *PMEL* (Supplementary Table S1) [28].

Four Colo679 MITF knockout (KO) clones were established. The expression of *GREB1 Is4* mRNA and protein was reduced in the MITF KO clones as well as *MLANA* and *PMEL* mRNAs (Fig. 2B). Reduced GREB1 Is4 expression by MITF knockdown (KD) was also confirmed in SKMEL28 and G361 cells (Supplementary Fig. S2C) and rescued by ectopic MITF expression in Colo679 cells (Supplementary Fig. S2D). Conversely, overexpression of wild-type (WT) MITF in non-melanoma 293T cells increased *GREB1 Is4*, *PMEL*, and *MLANA* mRNAs (Fig. 2C). An active form of MITF E318K, which prevents SUMOylation at K316 [29], induced these mRNAs expression more strongly than WT MITF (Fig. 2C).

*GREB1 Is4* transcription start site (TSS) and putative MITF-binding site were predicted by refTSS and ChIP atlas public data, respectively (Fig. 2D). There were three MITF-binding E-box CANNTG motifs within 0.3 kb of the 5’-upstream region of *GREB1* exon 19, including one CACTTG and two CAGCTG motifs [9]. Chromatin immunoprecipitation (ChIP) showed that MITF binding and H3K27 acetylation at this site (−55 to +47) in Colo679 cells, and their signals were not increased compared to control IgG at other *GREB1* locus (exon 24) (Fig. 2D).

To show the functional importance of this site, a reporter assay was performed. Because MITF-driven promoter activities of the sequence from −315 to 0 (TSS) covering MITF binding sites and the sequence from −82 to 0 including only two E-boxes were equally active (Supplementary Fig. S2E), the latter short sequence was used in this assay. Mutating either of the two E-boxes resulted in loss of MITF-mediated promoter activity (Fig. 2E and Supplementary Fig. S2F).

Analysis of our RNA-seq and ATAC-seq and public ChIP-seq data shows that the peaks of H3K27ac, MITF binding, and H3K4me3 were clearly observed at this site in melanoma cells, except for A375 cells (Fig. 2F). Moreover, the restricted *GREB1* expression in *MITF*-positive cells were confirmed by public single cell data sets. *GREB1* and *MITF* mRNAs were primarily expressed in melanocytic cells, but not in A375 cells or mesenchymal-like cells [24] (Supplementary Fig. S3A–C), and *GREB1* expression and NCSC-like state signatures appeared to be mutually exclusive [30] (Supplementary Fig. S3D–F). Thus, *GREB1 Is4* is a direct target of MITF and MITF specifically induces GREB1 Is4 expression in melanocytic melanoma cells.

### GREB1 is expressed in primary melanoma tumors

**Fig. 3:**
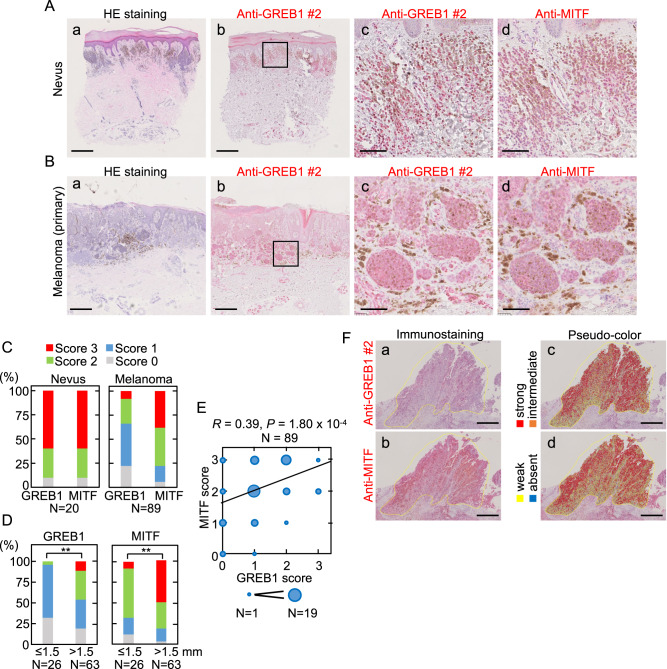
GREB1 and MITF expression is correlated with melanoma cases. **A** Serial sections of nevi were stained with hematoxylin-eosin (HE) (a), anti-GREB1(#2) (b, c) or anti-MITF (d) antibody. The boxed region in (b) was enlarged in (c). To distinguish from melanin pigment, the staining was visualized using the Warp Red Chromogen Kit, and sections counterstained with hematoxylin. Scale bars, 500 μm (a, b); 100 μm (c, d). **B** Primary melanoma tissues were stained with HE (a), anti-GREB1(#2) (b, c), or anti-MITF (d) antibody. The boxed region in (b) was enlarged in c. The staining was visualized as in **A**. Scale bars, 500 μm (a, b); 100 μm (c, d). **C**, **D** The distributions of the IHC scores (0–3) for GREB1 or MITF in the nevi and primary melanoma sections are shown as percentages (**C**). The melanoma samples were classified into two groups, depending on tumor thickness – smaller (*N* = 26) or larger (*N* = 63) than 1.5 mm. The distributions of the IHC scores for GREB1 or MITF are shown as percentages (**D**). **E** Scatter plots depict the relationship between the GREB1 and MITF scores in melanoma (*N* = 89). The size of the dots indicates the number of identical scores. The solid line indicates a linear fit. *R* and *P* represent the Pearson’s correlation coefficient and *P*-value, respectively. **F** Serial sections of a single melanoma specimen were stained with anti-GREB1(#2) (a) or anti-MITF antibody (b), and the respective staining intensities for individual cells were quantified with HALO soft and pseudo-colored into four levels: absent, weak, intermediate, and strong (c, d). Scale bars, 500 μm (a–d).

Expression of GREB1 and MITF in benign nevi (*N* = 20) and primary melanoma tumors (*N* = 89) was analyzed by immunohistochemistry (IHC) (Fig. 3A, B and Supplementary Fig. S4A, B). Staining was scored according to four categories (0–3) based on the staining intensity and area [31] (Supplementary Fig. S4C). Strong GREB1 or MITF staining (score 3) was observed in 12/20 (60.0%) benign nevus samples (Fig. 3C). In the nests of melanocytic nevus, co-staining of GREB1 and MITF and some scattered brown melanin pigments were observed (Figs. 3Ac, d). In the resected primary melanoma tumors, high expression of GREB1 (scores = 2 and 3) and MITF (score = 3) were observed in 30/89 (33.7%) and 34/89 (38.2%) tumors, respectively (Fig. 3C). MITF and GREB1 were strongly co-expressed in approximately 8/20 (40 %) of nevi and 17/89 (20%) of melanoma cases.

The clinicopathological parameters of melanoma cases with high GREB1 or MITF expression were compared to those with low GREB1 or MITF expression. The high GREB1 and MITF expression levels were significantly correlated with pathological T factor (pT), which reflects the tumor thickness. In contrast, there were no significant associations between GREB1 and MITF expression and age, sex, lymph node metastasis, melanoma subtypes, *BRAF* mutations, or stages (Supplementary Fig. S5A and Table 1). High MITF expression was significantly correlated with poor overall survival (Supplementary Fig. S5B). Although high GREB1 expression cases tended to exhibit a poor prognosis, the log-rank *P*-value was not statistically significant, likely due to the limited number of our cases (Supplementary Fig. S5B). The Skin-SKCM TCGA dataset (*N* = 474) showed that overall survival is significantly reduced in cases with high GREB1 or MITF expression compared to those with low expression (Supplementary Fig. S5C).

**Table 1:** Relationship between GREB1 and MITF expression and clinicopathological characteristics of malignant melanoma cases (*N* = 89).

Parameters	GREB1 high (*N* = 30)	GREB1 low (*N* = 59)	*P* value
Age (year)	66 (28–93)	70 (30–91)	0.811
Sex (male/female)	20/10	33/26	0.369
pT (Tis-2/3, 4)	3/27	29/30	0.0003
pN (0/1–3)	17/13	32/26	1.000
Subtypes (Acral/CSD/non-CSD/mucosal)	17/7/3/3	37/7/12/3	0.290
*BRAF* mutation (positive/negative)	2/10	1/12	0.593
pStage (0-II/III, IV)	17/13	35/24	0.824
ICI/MTT treated (treated/non-treated)	7/23	11/48	0.591

Parameters	MITF high (*N* = 34)	MITF low (*N* = 55)	*P* value
Age (year)	67 (28–93)	69 (30–86)	0.847
Sex (male/female)	24/10	29/26	0.121
pT (Tis-2/3, 4)	5/29	27/28	0.0013
pN (0/1–3)	15/19	36/19	0.077
Subtypes (Acral/CSD/non-CSD/mucosal)	20/7/4/3	34/7/11/3	0.559
*BRAF* mutation (positive/negative)	1/9	2/13	1.000
pStage (0-II/III, IV)	16/18	36/19	0.121
ICI/MTT treated (treated/non-treated)	10/24	8/47	0.108

*Tis* tumor in situ, *T1* tumor thickness is less than 1 mm, *T2* tumor thickness is more than 1.01 mm but less than 2.00 mm, *T3* tumor thickness is more than 2.01 mm, but less than 4 mm, *T4* tumor thickness is more than 4.0 mm, *N0* No metastatic Nodes, *N1* there is evidence of involvement of one regional lymph node, *N2* there is evidence of involvement of 2 to 3 regional lymph nodes, *N3* >4 metastatic nodes, or matted nodes, or in transit metastases/satellites with metastatic nodes, *M1* there is evidence of distant metastasis, *CSD* chronic sun-induced damage, *ICI/MTT* Immune checkpoint inhibitors/molecular targeted therapeutics.

Univariate analysis of in-house melanoma cases demonstrated that pStages III and IV and high MITF expression are significantly associated with shorter overall survival (Supplementary Table S2). Melanoma thickness is well correlated with the frequency of melanoma metastasis and an appropriate prognostic indicator for 5- or 10-year survival after excision of the primary tumor [32, 33]. High GREB1 and MITF expression levels were observed in 7/63 cases (11.1%) and 31/63 cases (49.2%), respectively, of primary melanoma with a depth over 1.5 mm. For melanoma cases with a depth of less than 1.5 mm, none had high GREB1 expression (0/26), while only 11.5% (3/26) of these cases had high MITF expression (Fig. 3D). Seven cases, in which GREB1 score is 3 and an invasion score is over 1.5 mm, showed that MITF score is 2 or 3. Melanoma cases with more than 1.5 mm depth showed poor overall survival (Supplementary Fig. S5B, C).

There was a positive correlation between the GREB1 and MITF IHC scores (0–3) among melanoma patients (Fig. 3E). In the serial sections of melanoma tissues expressing high GREB1 and MITF, the staining intensity for GREB1 and MITF was quantified and classified into four categories with pseudo-color (Fig. 3F). The results showed similar staining patterns, suggesting a correlation between GREB1 and MITF expression within the same cells. Thus, as well as MITF, GREB1 is expressed in benign nevi and tumor lesions of melanoma and their expression is correlated in both inter- and intra-tumors, and it is likely that GREB1 expression in tumor lesions is associated with aggressiveness of melanoma.

### GREB1 Is4 stimulates melanoma cell proliferation in vitro

**Fig. 4:**
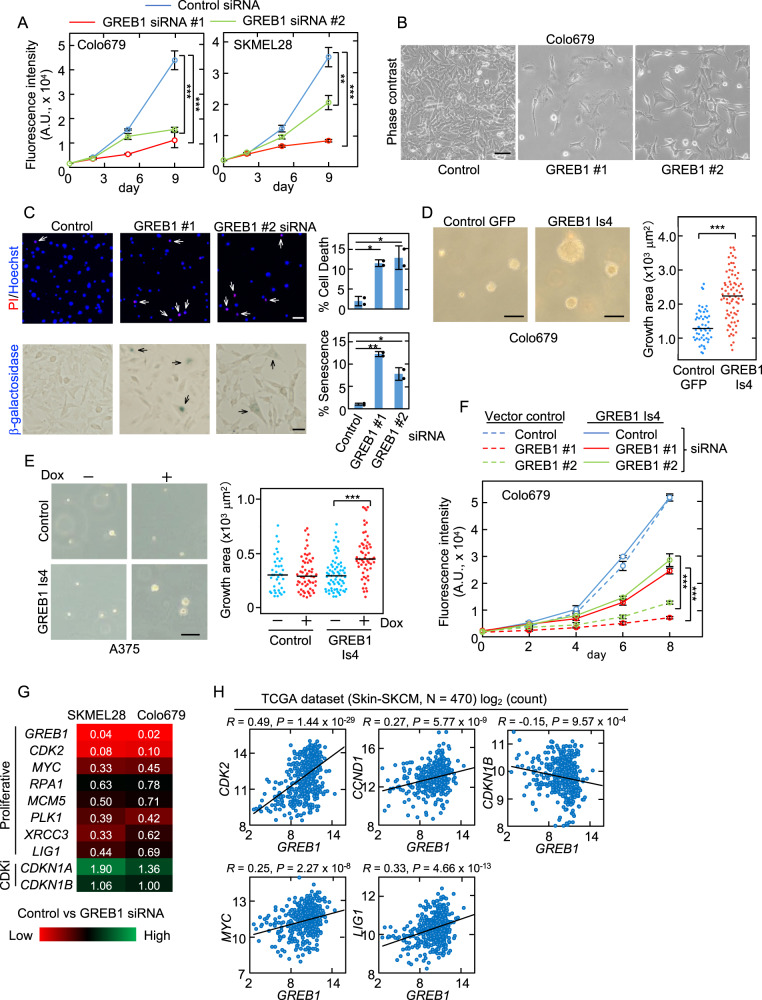
GREB1 Is4 stimulates melanoma cell proliferation in vitro. **A** Colo679 and SKMEL28 cells with 10 nM control or two GREB1 siRNAs were subjected to the 2D cell proliferation assay using CyQUANT NF. The data are presented as the mean ± s.d. from three independent experiments. **B** Morphology of Colo679 cells treated with control or GREB1 siRNAs. Scale bars, 50 μm. **C** Cell death or senescence of Colo679 cells with control or two GREB1 siRNAs were visualized with PI/Hoechst33342 or β-galactosidase staining, respectively. The white and black arrows indicate dead and senescent cells, respectively. The positive cells are shown as the percentage to total cells per field (*n* = 100–200). The data are presented as the mean ± s.d. from two independent experiments. Scale bars, 100 μm (top); 50 μm (bottom). **D** Colo679 cells expressing GFP or GREB1 Is4 were plated in soft agar. After 14 days, the colony areas were determined. The distribution was visualized using a bee swarm plot. The bars show the median of the colony areas. Scale bars, 60 μm. **E** A375 cells expressing Dox-inducible GREB1 Is4 or vector control were plated in soft agar with or without Dox 20 ng/ml. GREB1 Is4 induction was checked (Supplementary Fig. S6E). After 7 days, the colony areas were determined and shown as above. Scale bar, 50 μm. **F** Colo679 cells expressing vector or GREB1 Is4 were transfected with control or two GREB1 siRNAs and subjected to the 2D cell proliferation assay. The data are presented as the mean ± s.d. from three independent experiments. In **A** and **C**–**F**, (*P < 0.05; **P < 0.01; ***P < 0.001, Student’s *t* test). **G** SKMEL28 and Colo679 cells were transfected with 10 nM control or GREB1 #1 siRNA for 48 h. The mRNA expression was analyzed by qPCR. The data were normalized to *GAPDH* and expressed as fold-change (white number), relative to the control siRNA. The results were standardized by min-max normalization and are presented as a heat map. **H** The scatter plots indicate the correlation between the mRNA levels of *GREB1* (x-axis) and proliferative (*CDK2, MYC, CCND1, LIG1*) and CDK inhibitor (*CDKN1B*) genes (y-axis) in TCGA Skin-SKCM. The solid lines indicate a linear fit. *R* and *P* represent the Pearson’s correlation coefficient and *P*-value, respectively.

To examine the role of GREB1 Is4 in melanoma cell proliferation, GREB1 Is4 was knocked down in Colo679 and SKMEL28 cells using two different siRNAs (Supplementary Fig. S6A). GREB1 Is4 KD decreased cell proliferation in 2-dimensional (2D) assay (Fig. 4A). GREB1 siRNA-treated cells were larger and more flattened, and some cells were detached from the dish and rounded (Fig. 4B), and about 10% cells died and became senescent (Fig. 4C). In contrast, these siRNAs did not affect the proliferation of A375 or MM-RU cells, which do not express GREB1 (Supplementary Fig. S6B). GREB1 Is4 KD by two different GREB1 antisense oligonucleotides (ASO) also decreased cell proliferation of Colo679 cells (Supplementary Fig. S6C). Conversely, GREB1 Is4 overexpression increased the size of colonies formed by Colo679 and A375 cells in the soft agar (Fig. 4D, E and Supplementary Fig. S6D, E).

Furthermore, ectopic GREB1 Is4 expression also rescued the growth inhibition by GREB1 Is4 KD in Colo679 and MeWo cells (Fig. 4F and Supplementary Fig. S6F–H). Ten Colo679 GREB1 Is4 KO clones were established using CRISPR-Cas9 gene editing (Supplementary Fig. S7A). GREB1 Is4 KO did not affect MITF expression (Supplementary Fig. S7A). The proliferation of the GREB1 Is4 KO clones was less than that of the control cells, while the proliferation of the MITF KO clones was severely defective (Supplementary Fig. S7B). The reasons for the difference of the phenotype between GREB1 KO and MITF KO clones may be due to that the clones, which proliferate without senescence and cell death under the conditions of GREB1 KO, were selected and that many downstream genes other than GREB1 Is4 were reported as MITF target genes. DepMap data from the online open-access “cancer dependency map,” which systematically identifies the genetic dependency of hundreds of cell lines, also showed that skin cancer was sensitive to *GREB1* and *MITF* genetic perturbations (Supplementary Fig. S7C). Colo679 cells were the most vulnerable to GREB1 RNAi of all the cell lines tested, whereas A375 cells were not affected (Supplementary Fig. S7C). Thus, GREB1 Is4 is necessary for melanocytic melanoma cell proliferation.

Gene expression profile was examined in GREB1 Is4 KD SKMEL28 and Colo679 cells using qPCR. Among several marker genes were selected [9, 34, 35], the expression of proliferative marker genes (e.g., *CDK2*, *MYC*, and *LIG1*) decreased, whereas the expression of *CDKN1A* (cyclin-dependent kinase inhibitor) increased (Fig. 4G). GREB1 Is4 overexpression rescued the expression of proliferative and CDKi genes in GREB1 Is4 KD Colo679 cells (Supplementary Fig. S7D). Similarly, analysis of the melanoma TCGA dataset revealed that *GREB1* gene expression was positively correlated with the expression of proliferative marker genes (*CDK2*, *MYC*, *CCND1*, and *LIG1*) and negatively correlated with *CDKN1B* (Fig. 4H). Taken together, these results suggest that GREB1 Is4 regulates melanoma cell proliferation in association with expression changes of cell proliferation-related genes.

### GREB1 Is4 promotes melanoma proliferation in vivo

**Fig. 5:**
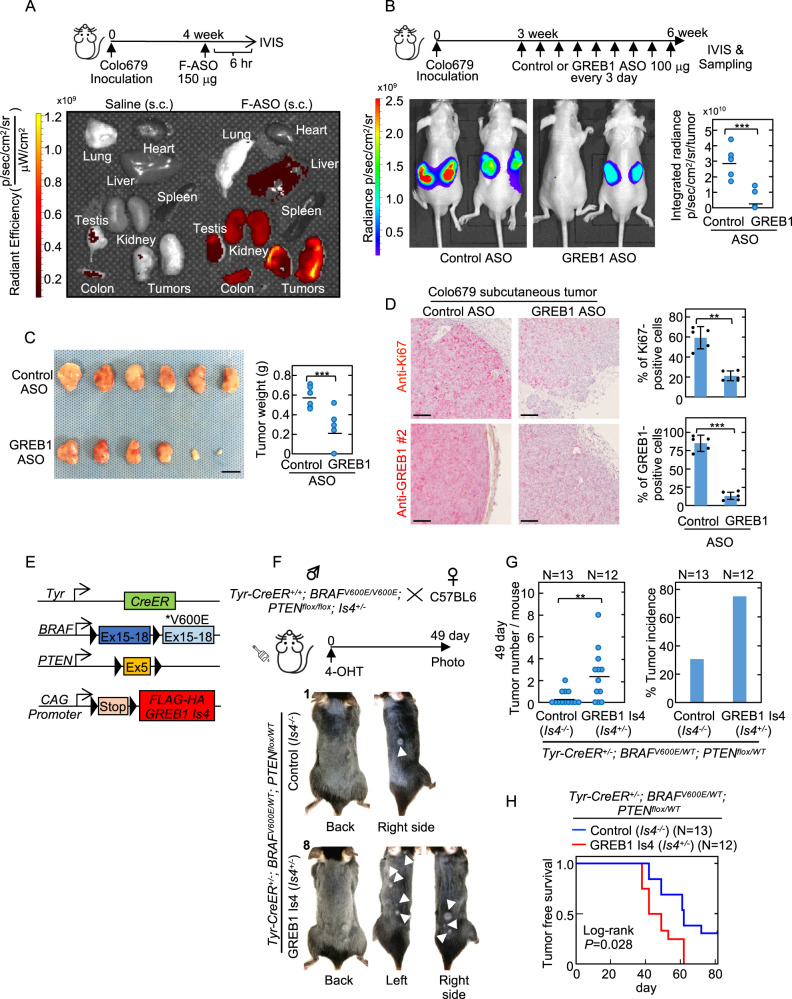
GREB1 Is4 promotes melanoma proliferation in vivo. **A** Colo679 cells (2.5 × 10^6^ cells) were subcutaneously implanted into the dorsal flank of nude mice on day 0. After four weeks, mice were subcutaneously injected with saline or 6-carboxyfluorescein (FAM)-ASO (150 μg). After 6 h, the mice were euthanized, and the fluorescence intensities in the various organs were measured using the IVIS imaging system. **B** Colo679 cells (2.5 × 10^6^ cells) in 50% Matrigel were subcutaneously implanted into the dorsal flank of nude mice (*N* = 6 per group) on day 0. After three weeks, the tumor-bearing mice received subcutaneous injections of 100 μg control ASO or GREB1 ASO-7724 every three days. IVIS images are shown for subcutaneous tumors six weeks after inoculation. The major integrated radiance is presented in dot plots (***P < 0.001, Student’s *t* test). **C** Nude mice bearing the melanoma tumors used in **B** were euthanized six weeks after tumor inoculation. Images of the resected subcutaneous tumors are shown. The weights of the tumors were measured and presented in dot plots (***P < 0.001, Student’s *t* test). Scale bar, 1 cm. **D** The xenograft tumors from **C** were fixed with paraformaldehyde. Tumor sections were stained with anti-Ki-67 or anti-GREB1 (#2) antibody. The GREB1- or Ki-67-positive cells were counted by HALO software. The data are presented as the percentage of positively stained cells compared to the total number of cells (**P < 0.01; ***P < 0.001, Student’s *t* test). Scale bars, 100 μm. **E** The schema of the *Tyr-CreER, BRAF*^*V600E*^*, PTEN*^*flox*^ and *GREB1 Is4* allele is shown. **F** A schematic representation of melanoma induction in control and GREB1 Is4 mice is presented. A 4-OHT solution was applied topically to the whole back of neonatal mice on days 2–6 after birth. 49 days after 4-OHT administration, representative photographs were taken for the shaved dorsal skin of control and GREB1 Is4 melanoma mice. The white arrow heads indicate melanoma nodules. The number in the upper left of the picture indicate the number of tumor nodules. **G** The number of tumor nodules per mouse and the percentage of the tumor incidence at 49 days are shown (*N* = 12–13 mice per genotype). **H** Tumor-free survival rates were compared between control (blue) and GREB1 Is4 (red) melanoma mice. The log-rank test was used for statistical analysis.

To examine whether GREB1 Is4 is involved in melanoma proliferation in vivo, luciferase-expressing Colo679 cells were injected subcutaneously into immunodeficient mice, and the effects of GREB1 Is4 antisense oligonucleotides (ASO) were evaluated by administering the ASO in the distant subcutaneous region. The highest fluorescence intensities of 6-FAM-ASO measured using the IVIS Imaging system were detected in the tumors compared to the organs (e.g., liver and kidney), suggesting that systemically injected ASO is likely to accumulate in tumors (Fig. 5A). The fluorescence intensities and weights of the tumors from mice treated with GREB1 ASO-7724 decreased compared to mice treated with control ASO (Fig. 5B, C). GREB1 ASO-7724 also decreased the number of Ki-67- and GREB1-positive cells (Fig. 5D). Thus, GREB1 is involved in melanocytic melanoma growth in vivo.

To further confirm the role of human GREB1 Is4 in vivo using another mouse model, conditional transgenic (Tg) mice were generated with inducible GREB1 Is4 expression (*Is4*^+/−^ mice) (Fig. 5E and Supplementary Fig. S8A, B). *Tyr-CreER; BRAF*^*V600E*^*; PTEN* ^*flox*^ melanoma mice were crossed with the *Is4*^+/−^ mice, generating control (*Tyr-CreER; BRAF*^*V600E*^*; PTEN* ^*flox*^*; Is4*^−/−^) and GREB1 Is4 (*Tyr-CreER; BRAF*^*V600E*^*; PTEN*^*flox*^*; Is4*^+/−^) mice (Fig. 5E). Induction of FLAG-HA hGREB1 Is4 protein in tumors from GREB1 Is4 melanoma mice was confirmed by western blotting (WB) and IHC (Supplementary Fig. S8C, D).

*Tyr-CreER*^*+/+*^*; BRAF*^*V600E/V600E*^*; PTEN*^*flox/flox*^*; Is4*^+/−^ and WT mice were crossed, and 4-OHT was applied to the entire back of their newborns, and the effect of GREB1 Is4 expression on melanoma development was evaluated using littermates. Approximately 30% of control mice developed tumors within 49 days after 4-OHT treatment, whereas over 70% of GREB1 Is4 mice developed tumors (Fig. 5F, G). Tumor-free survival of GREB1 Is4 mice was reduced compared to control mice (Fig. 5H). No tumor formation in the presence of PTEN (*Tyr-CreER*^*+/+*^*; BRAF*^*V600E/V600E*^*; PTEN*^*WT/WT*^*; Is4*^+/−^) was observed within five months after GREB1 Is4 induction (data not shown). These results demonstrated that GREB1 Is4 expression accelerates melanoma formation in the *BRAF*^*V600E*^*; PTEN* ^*flox*^ melanoma mouse model.

No mouse *Greb1* gene expression was observed in the tissues and cell clone (Yummer1.7) from the mouse *BRAF*^*V600E*^*; PTEN* ^*flox*^ melanomas nor in B16BL6 and B16F10 mouse melanoma cell lines expressing MITF target gene (*Pmel*) (Supplementary Fig. S8E). Consistently, mouse GREB1 isoforms equivalent to human GREB1 Is4 were not reported in the Ensembl data (Supplementary Fig. S8F), indicating that GREB1 Is4 expression is specific to human melanoma.

### CAD is identified as a GREB1 Is4-binding protein

**Fig. 6:**
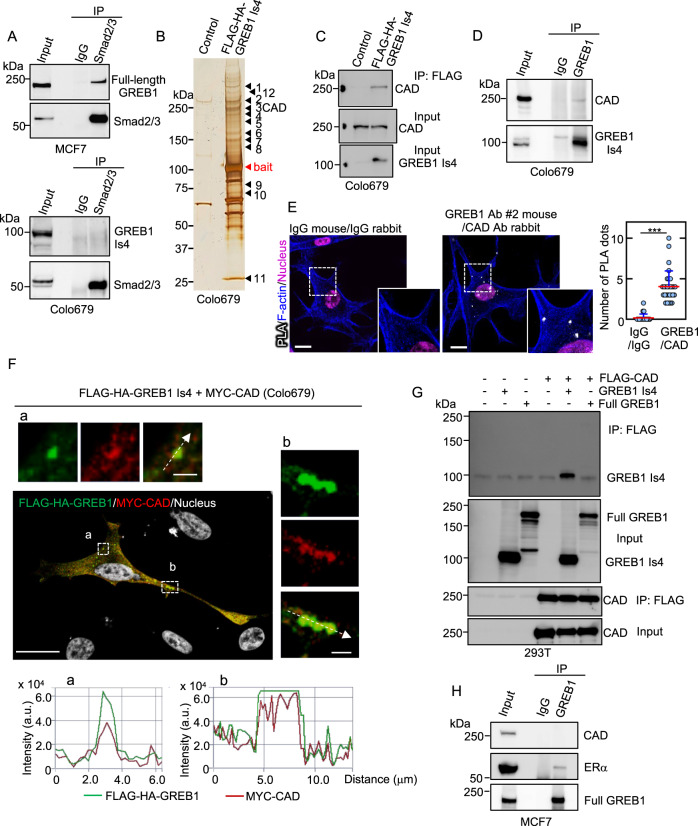
CAD is identified as a GREB1 Is4-binding protein. **A** Lysates of MCF7 and Colo679 cell were immunoprecipitated with anti-Smad2/3 antibody, and the immunoprecipitates were probed with anti-GREB1(#2) or anti-Smad2/3 antibody. **B** Lysates from Colo679 cells expressing GFP (control) or FLAG-HA-GREB1 Is4 were immunoprecipitated with an anti-FLAG antibody. The immunoprecipitates were eluted using a FLAG peptide, and FLAG-HA-GREB1 Is4-interacting proteins were detected by silver staining and subjected to LC-MS/MS analysis. FLAG-HA-GREB1 Is4 (bait) and CAD are indicated by a red arrowhead and No.3 band, respectively. The other identified bands are listed in Supplementary Table S3. **C** Colo679 cell lysates were immunoprecipitated with control IgG and anti-FLAG antibody, and the immunoprecipitates were probed with anti-CAD or anti-GREB1(#2) antibody. **D** Lysates from Colo679 cells were immunoprecipitated with an anti-GREB1(#3) antibody and the immunoprecipitates were probed with anti-CAD or anti-GREB1(#2) antibody. **E** Colo679 cells were incubated with mouse anti-GREB1(#2) and rabbit anti-CAD antibodies, which were then combined with secondary PLA probes. Interaction events are shown as white dots. Cells with PLA dots were counted, and the results are expressed as the percentage of total cells per field (*n* > 100) (***P < 0.001 by the Student’s *t* test). The regions in the solid squares are shown enlarged. Phalloidin or Hoechst33342 was used as a F-actin or nucleus staining marker, respectively. Scale bars, 10 μm. **F** Colo679 cells expressing FLAG-HA-GREB1 Is4 and MYC-CAD were stained with the anti-FLAG and anti-MYC antibodies, and counterstained with Hoechst33342. Scale bar, 20 μm. The boxed region in a and b was enlarged to show monochromatic stained images above and to the right, respectively. Scale bar, 2 μm. The fluorescence intensities of FLAG-HA-GREB1 Is4 (green) and MYC-CAD (red) at the white arrow sections are shown in a and b below. **G** Lysates from 293T cells expressing FLAG-CAD and full-length GREB1 or GREB1 Is4 were immunoprecipitated with anti-FLAG antibody, and the immunoprecipitates and input lysates were probed with the indicated antibodies. **H** Lysates from MCF7 cells were immunoprecipitated with an anti-GREB1 (#3) antibody and the immunoprecipitates were probed with anti-CAD, anti-ERα, or anti-GREB1(#2) antibody.

GREB1 interacted with Smad2/3 in MCF7 cells, consistent with previous observations in HepG2 cells [15]; however, GREB1 Is4 did not form a complex with Smad2/3 in Colo679 cells (Fig. 6A). To clarify the mechanism by which GREB1 Is4 stimulates cell proliferation, FLAG-HA GREB1 Is4 was expressed in Colo679 and used as a bait to screen for its binding proteins (Fig. 6B and Supplementary Table S3). GREB1 Is4-interacting proteins were also analyzed in other two different cell lines, including HeLa and U2OS cells (Supplementary Fig. S9A and Supplementary Tables S4, S5). Among possible candidate proteins, CAD was the only protein that commonly bound to GREB1 Is4 in all three cell lines.

CAD is a trifunctional multi-domain enzyme involved in the first three steps of pyrimidine biosynthesis and contains GLNase (glutaminase), CPSase (carbamoyl-phosphate synthetase), DHOase (dihydroorotase), and ATCase (aspartate transcarbamylase) functional domains [19, 20]. Binding of FLAG-HA GREB1 Is4 to endogenous CAD was confirmed by immunoprecipitation in Colo679 cells (Fig. 6C). Endogenous GREB1 Is4 immunoprecipitated endogenous CAD in Colo679 cells (Fig. 6D), and proximity ligation assay (PLA) also confirmed the endogenous interaction between GREB1 Is4 and CAD in the cytoplasm (Fig. 6E). When either FLAG-HA-GREB1 Is4 (green) or Myc-CAD (red) was overexpressed, they were present throughout the cytoplasm (Supplementary Fig. S9B). When they were expressed simultaneously, co-localization of both proteins was observed as punctate staining and quantified by line plot quantification (Fig. 6F). In addition, GREB1 Is4 but not full-length GREB1 was immunoprecipitated with FLAG-CAD in 293T cells (Fig. 6G), and CAD was not immunoprecipitated with full-length GREB1 in MCF7 cells under the condition that GREB1 associated with endogenous ERα (Fig. 6H). Thus, GREB1 Is4, but not full-length GREB1, specifically binds to CAD. Further analysis revealed that FLAG-HA-GREB1 Is4 forms a complex with the CPSase domain of CAD (Supplementary Fig. S9C).

### GREB1 Is4 is involved in de novo pyrimidine synthesis

**Fig. 7:**
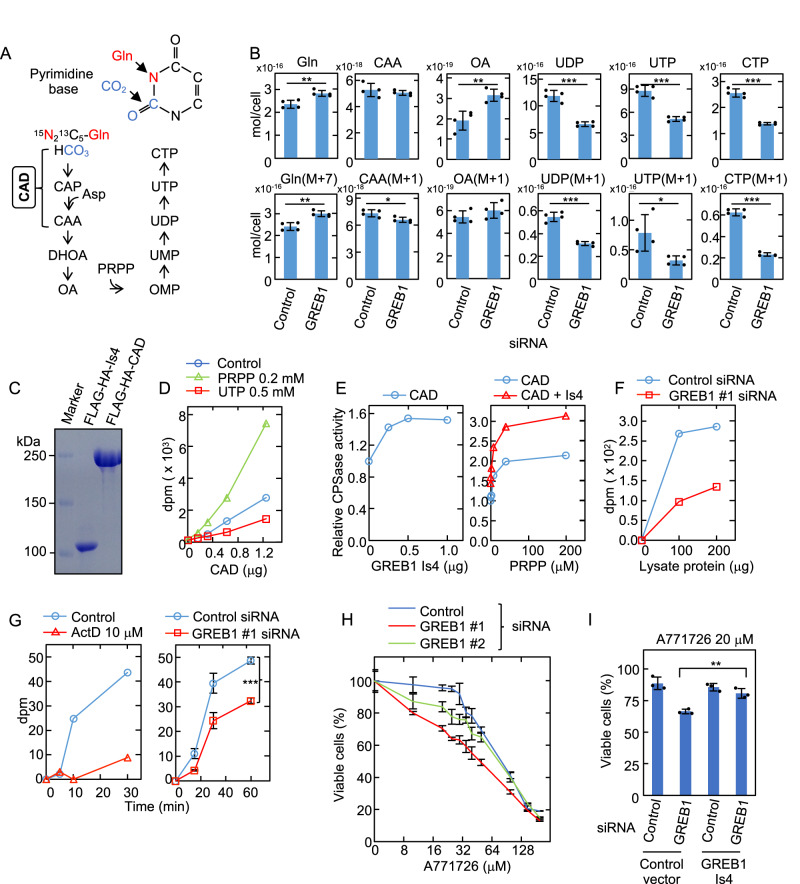
GREB1 Is4 is involved in de novo pyrimidine synthesis. **A** The schematic diagram depicts the de novo pyrimidine synthesis pathway and the pyrimidine base ring incorporating the nitrogen and carbon atoms from glutamine and CO_2_, respectively. CAP carbamoyl phosphate, CAA carbamoyl aspartic acid, DHOA dihydroorotate, OA orotate, OMP orotidine monophosphate. **B** Colo679 cells transfected with control or GREB1 #1 siRNA for 60 min were labeled with ^15^N_2_^13^C_5_-glutamine, and the intracellular concentrations of the indicated metabolite concentrations were measured using IC- and LC-QEMS analysis. Experiments were performed in quadruplicate and the data are presented as the mean ± s.d. (*P < 0.05; **P < 0.01; ***P < 0.001, Student’s *t* test). **C** FLAG-HA-GREB1 Is4 and FLAG-HA-CAD were purified from 293T cells and subjected to SDS-PAGE followed by CBB staining. **D** The CPSase activity of FLAG-HA-CAD in the presence or absence of 0.2 mM PRPP or 0.5 mM UTP. The results shown are representative of three independent experiments. **E** Left panel, the CPSase activity of purified FLAG-HA-CAD (0.5 μg) in the presence of the indicated amounts of purified FLAG-HA-GREB1 Is4. Right panel, the CPSase activity of FLAG-HA-CAD Is4 (0.5 μg) and FLAG-HA-GREB1 (0.5 μg) with the indicated concentrations of PRPP. The results are shown as the fold increase. **F** The CPSase activity in lysate from Colo679 cells treated with control or GREB1 #1 siRNA. **G** Left panel, Colo679 cells were labeled with 4.3 mM ^14^C-labeled sodium bicarbonate (74 kBq) in the presence or absence of 10 μM actinomycin D (ActD) (transcriptional inhibitor) for the indicated times. The incorporated radioactivity into total RNA was counted with a liquid scintillation counter. ActD was used for positive control of this assay. Right panel, the incorporated radioactivity into total RNA from Colo679 cells transfected with control or GREB1 siRNA was counted as described above. The data are presented as the mean ± s.d. from three independent experiments (***P < 0.001, Student’s *t* test). **H** The equal number of Colo679 cells transfected with control or two GREB1 siRNAs was treated with the indicated concentrations of A771726 (DHODH inhibitor) for 4 days. Cell viability was measured by the CellTiter-Glo kit. The results are shown as a percentage compared to those of cells treated with vehicle (DMSO) only, and expressed as the mean ± s.d. of three independent experiments. X axis is described in log scale. **I** Colo679 cells expressing control vector or exogenous GREB1 Is4 were treated with control or GREB1 #1 siRNA for 2 days and then the equal number of Colo679 cells was incubated with 20 μM A771726 for 4 days and cell viability was measured as described in **H**. The results are shown as a percentage compared to those of cells treated with vehicle (DMSO) only, and expressed as the mean ± s.d. of three independent experiments. **P < 0.01, Student’s *t* test.

Flux analysis was used to clarify the role of GREB1 Is4 in de novo pyrimidine nucleotide biosynthesis using a 60-min pulse labeling of ^15^N_2_, ^13^C_5_-glutamine, of which one nitrogen atom is incorporated into the pyrimidine base ring (Fig. 7A). After the pulse, the intracellular concentrations of the pyrimidine metabolites were measured using ion chromatography-quadrupole electrophoresis mass spectrometry (IC-QEMS). The levels of CAA (M + 1) showed statistically significant decrease in the GREB1 Is4 KD cells; the reductions in UDP (M + 1), UTP (M + 1), and CTP (M + 1) by GREB1 Is4 KD were also shown (Fig. 7B). The upregulation of intracellular Gln and Gln(M + 7) concentrations was observed upon GREB1 silencing, probably due to the reduction in Gln consumption. In addition, the same analysis was performed using metabolites (M + 2) extracted from the IC-QEMS data. When CAP was metabolized to CAA, ^15^N of aspartic acid (^15^N is supplied to Asp by the Gln->Glu->Asp pathway) was added and metabolites of the pyrimidine pathway also included two ^15^N (M + 2) except for CAP (Supplementary Fig. S10A). The similar results were obtained when labeled (M + 2) was calculated (Supplementary Fig. S10B). Furthermore, when the ratio of labeled (M + 1 + M + 2) to total metabolites (M + 0 + M + 1 + M + 2) in each pyrimidine metabolite was calculated, CAA, OA and CTP were decreased (Supplementary Fig. S10C). Moreover, we conducted a comprehensive analysis of the metabolome using CE-MS, which revealed that the metabolic pathway most significantly impacted by GREB1 silencing was pyrimidine metabolism (Supplementary Table S6). However, it was noteworthy that other metabolic pathways, such as arginine biosynthesis and purine metabolism, were also affected. Consistently, when pyrimidine metabolites (specifically uridine, thymidine, and cytidine) were added to the culture medium of GREB1 knockdown cells, pyrimidine metabolites did not alleviate the inhibition of cell proliferation caused by GREB1 silencing (Supplementary Fig. S10D), indicating that pyrimidine synthesis alone is insufficient to rescue the impairment of cell proliferation. Taken together, these results suggested that GREB1 Is4 is primarily required for de novo pyrimidine synthesis in melanoma cells and has other regulatory roles for cell proliferation.

To evaluate the direct effect of GREB1 Is4 on CPSase activity of CAD, FLAG-HA GREB1 Is4 and FLAG-HA CAD were purified (Fig. 7C). The CPSase activity is allosterically inhibited by UTP, an end product, and activated by PRPP (phosphoribosyl diphosphate), a sugar substrate for pyrimidine and purine biosynthesis [36]. When purified CAD was incubated with ^14^C-labeled sodium bicarbonate, glutamine, and aspartate in the presence or absence of UTP or PRPP, CPSase activity was indeed inhibited by UTP and promoted by PRPP (Fig. 7D). GREB1 Is4 enhanced the CPSase activity (Fig. 7E, left panel). Moreover, the CPSase activation by PRPP was further enhanced by GREB1 Is4 (Fig. 7E, right panel). Colo679 cell lysates had CPSase activity, which was reduced in lysates from GREB1 Is4 KD Colo679 cells (Fig. 7F). De novo RNA synthesis was measured with the assessment of ^14^C-labeled sodium bicarbonate incorporation into total RNA of Colo679 cells. Newly synthesized RNA was also reduced in GREB1 Is4 KD cells (Fig. 7G), suggesting that GREB1 Is4 plays a direct role in pyrimidine biosynthesis through the control of CAD CPSase activity.

Finally, to examine the role of GREB1 Is4-dependent pyrimidine metabolism in melanoma cell proliferation, the effects of A771726 (DHODH inhibitor) [37] on cell viability in GREB1 Is4 KD melanoma was examined. GREB1 siRNA-treated Colo679 cells were more sensitive to A771726 than control siRNA-treated cells (Fig. 7H) and exogenous GREB1 Is4 expression restored its sensitivity (Fig. 7I), indicating that GREB1 Is4 is necessary for resistance to A771726. Therefore, GREB1 Is4 expression may be involved in melanocytic melanoma cell proliferation via at least the regulation of pyrimidine metabolism.

## Discussion

Although treatment and survival of melanoma patients have improved dramatically in the past ten years, half of advanced melanoma cases are still resistant to current therapies [35, 38, 39]. GREB1 is believed to be involved in hormone-dependent cancer growth, but it has recently been shown that GREB1 expression is also associated with hepatoblastoma, a hormone-independent tumor [15]. The current study provided another example of GREB1 expression associated with a hormone-independent tumor, namely malignant melanoma. GREB1 has at least seven isoforms, each transcribed from unique promoters with divergent 5’ untranslated regions. GREB1 Is4 transcription starts from exon 19 of *GREB1*, and MITF directly controlled GREB1 Is4 expression in melanocytic melanoma. Indeed, MITF and GREB1 Is4 were co-expressed in melanocytic melanoma cells lines and in 40% of benign nevi, and 20% of melanoma tumors. Functionally, GREB1 Is4 expression stimulated melanoma cell proliferation. Consequently, targeting GREB1 offers a new and unique approach to inhibit melanoma growth.

Candidate cancer genes that induce melanoma with the *BRAF*^V600E^ mutation were screened using *Sleeping Beauty* (SB) transposon-mediated insertional mutagenesis in mice, and GREB1 was predicted as one of these genes [40]. Importantly, the SB transposon cassette was inserted in the forward orientation into the *GREB1* locus and all of which were upstream of mouse *greb1* exon 19, a position that allows transcriptional activation, including the exon corresponding to human GREB1 Is4 (Supplementary Fig. S11), indicating that it is a proto-oncogene, whereas the SB transposon was inserted in the reverse orientation into the *CDKN2A* and *PTEN* loci, indicating that they are tumor suppressor genes [40]. When GREB1 Is4 was expressed in the neonatal stage of *BRAF*^*V600E*^*; PTEN*^*flox*^ mice, it promoted the formation of *BRAF*^*V600E*^*; PTEN*^*flox*^ melanoma. The *MITF*^*E318K*^ mutation accelerates melanoma formation in the same background mice [41], and loss of MITF in *BRAF*^*V600E*^ zebrafish melanoma mutants leads to melanoma regression [42]. Because MITF is essential for melanoma development and GREB1 expression, GREB1 may mediate MITF-dependent melanomagenesis.

We found that GREB1 Is4 is localized to the cytosol of cells and binds to CAD in melanoma cells, resulting in positive cooperation for pyrimidine biogenesis. Moreover, pyrimidine biosynthesis is a promising synthetic lethal target for the *BRAF*^*V600E*^ mutation in melanoma [37]. Leflunomide, a DHODH inhibitor, was identified as an inhibitor of neural crest cell development by chemical screening, and A771726 also decreased melanoma cell growth in combination with GREB1 Is4 targeting (Fig. 7H). Although we could not exclude that decreased pyrimidine synthesis is an indirect effect of cell growth inhibition, GREB1 Is4 directly stimulated CAD CPSase activity in vitro, suggesting that GREB1 Is4 is required for de novo pyrimidine synthesis through CAD. GREB1 Is4 would contribute to the formation and/or survival of tumors in melanocytic melanoma because pyrimidine synthesis pathway is essential for various cellular functions.

GREB1 ASO was delivered mostly to melanoma tumors following systemic administration and reduced tumor size in a xenograft mouse model. In the melanoma TCGA dataset, the *GREB1* expression levels did not differ between patients with and without *BRAF* mutation, and even in WT *BRAF* patients, who would be expected to be ineffective on BRAF inhibitors, the GREB1 high group had a poor prognosis (Supplementary Fig. S12A, B). In addition, in the CD8A low expression group, where T cell infiltration was low and immune checkpoint inhibitors were not expected to be effective, there was a difference in prognosis depending on GREB1 expression, but not in the CD8A high expression group (Supplementary Fig. S12C). Because the targeting of GREB1 Is4 could inhibit melanocytic melanoma growth in a different way from BRAF or immune checkpoint inhibitors, it paves the way for new clinical trials to eradicate malignant melanoma.

## Materials and methods

### Materials and chemicals

HepG2 cells were purchased from the American Type Culture Collection (ATCC, Manassas, VA, USA). MCF7, G-361, MeWo, SKMEL28, MMRU, and Colo679 cells were from the Japanese Collection of Research Bioresources (JCRB, Osaka, Japan). A375 cells were from the KAC Co. (Amagasaki, Japan). Lenti-X^TM^293T (293T) cells were obtained from Takara Bio, Inc. (Shiga, Japan). HepG2 cells were grown in Eagle’s minimum essential medium supplemented with 10% fetal bovine serum (FBS), nonessential amino acids (NEAA), and GlutaMAX. 293T, G-361, MeWo, SKMEL28, A375, MM-RU, and Colo679 cells were grown in Dulbecco’s modified Eagle’s medium (DMEM) supplemented with 10% FBS. MCF7 cells were grown in DMEM supplemented with 10% FBS, NEAA, and 1 mM sodium pyruvate. The cell lines were tested monthly for mycoplasma using the LookOut Mycoplasma PCR Detection Kit (Sigma Aldrich, MP0035, St Louis, MO, USA). Cell line identity was confirmed using STR profiling.

### RNA and ATAC sequences of human melanoma samples

Patient melanoma tumors were shredded into 5-mm squares and implanted subcutaneously into NSG mice (Charles River, Yokohama, Japan). Grown tumors were harvested and transplanted into NOD-SCID mice (Charles River, Yokohama, Japan). Tumors were removed from the transplanted mice, and the human cells were recovered using MojoSort (Biolegend, San Diego, CA, USA). RNA was extracted from these cells, and an RNA-seq library was prepared as previously described [43]. An ATAC-seq library was also prepared using the omniATAC-seq method [44]. Sequencing was performed using Illumina Hiseq X.

### Open source data analysis

Gene expression TCGA data for tumor patients and GTEx data were obtained from the R2: genomics analysis and visualization platform (http://r2.amc.nl) and the UCSC Xena site (http://xena.ucsc.edu), respectively. All gene expression data, *P*-values, and *R*-values were downloaded. RNA-seq data of A375 cells (GSM1138787) was derived from GEO datasets (https://www.ncbi.nlm.nih.gov/geo/). *GREB1 Is4* TSS was predicted using refTSS (http://reftss.clst.riken.jp/reftss/TSS:Hg_18909.1). Public ChIP data for H3K27ac in A375 (SRX2267496) and Colo679 (SRX1451224), MITF in A375 (SRX8257422) and Colo829 (SRX346924), RNA Pol II in A375 (SRX8257400), H3K4me3 in A375 (SRX2896727) and PDX melanoma (SRX1136744) were obtained from the ChIP-Atlas data (https://chip-atlas.org/). MITF binding site at the *GREB1 Is4* promoter region were predicted from the ChIP Atlas database of Colo829 cells (ID: SRX346924).

### Patients and clinical specimens

Tissue samples were obtained with informed consent from 89 newly diagnosed melanoma patients (median age 68 years, range 28–93 years) who underwent surgical resection at Osaka University Hospital between February 1991 and August 2019. Approval for the use of human tissues was obtained from the Ethical Review Board of the Graduate School of Medicine, Osaka University, Japan.

### Generation of the mouse melanoma model

Mouse experiments were performed at the Institute of Animal Sciences Faculty of Medicine, Osaka University. The inducible mouse melanoma model was obtained by crossing the *Tyr-CreER* (donated by Dr. M Bosenberg [45]), *PTEN*^*flox*^ (donated by Dr. Tak W Mak [46]), *BRAF*^*V600E*^ (donated by Dr. M McMahon [47]), and GREB1 Is4 conditional Tg mouse strains to generate the experimental *Tyr-CreER; BRAF*^*V600*^*; PTEN*^*flox*^*; Is4*^+/−^ mice. For Cre induction, 12.5 mg/mL 4-hydroxytamoxifen (4-OHT, Nacalai tesque, Kyoto, Japan) in DMSO was topically applied to the entire mouse back on postnatal days (P) 2–6 neonates once a day on two consecutive days using a paintbrush. Endpoint of tumor free survival experiments was at 80 days and the experiment was stopped when one melanoma nodule was observed. Survival tumor free curves were determined using Kaplan-Meier analysis.

### Isolation of GREB1 Is4-interacting proteins

Briefly, Colo679 cells stably expressing FLAG-HA-GREB1 Is4 were lysed with NP-40 buffer. After centrifugation, M2 agarose beads were incubated with the supernatant and washed with NP-40 buffer. The GREB1 Is4–interacting proteins were eluted with FLAG peptide (0.5 mg/mL) and detected by silver staining. The candidate bands were cut from the gel, and the proteins were identified by trypsin digestion followed by LC-MS/MS analysis. Details of the method are described in the supplementary information.

### Quantitative RT-PCR (qPCR)

Total RNA was isolated using the NucleoSpin RNA kit (TAKARA, Shiga, Japan). Total RNA (1 μg) was reverse transcribed to cDNA with ReverTra Ace qPCR RT Master Mix (TOYOBO, Osaka, JAPAN), according to the manufacturer’s instructions. qPCR was performed using the Applied Biosystems StepOne Real-time PCR System (Waltham, MA, USA). The forward and reverse primers are listed in Supplementary Table S7.

### Statistical analysis

Student’s *t* test was used to determine if there was statistical difference between the means of two groups. Statistical analysis was performed using Excel 2016 and GraphPad Prism 7 (GraphPad Software, La Jolla, CA, USA). *P*-values < 0.05 were considered statistically significant.

### Study approval

The protocols for the use of human specimens in immunohistochemistry and RNA and ATAC sequences were approved by the Ethical Review Board of the Graduate School of Medicine, Osaka University, Japan (No. 13455, 709, and 1501902), following the Declaration of Helsinki. All studies were performed in accordance with the Committee guidelines and regulations. Written informed consent was obtained from all patients. All protocols used for the animal experiments for this study were approved by the Animal Research Committee of Osaka University, Japan (No. 21-032-048).

### Supplementary information

Supplementary Information

Supplementary Figure1

Supplementary Figure2

Supplementary Figure3

Supplementary Figure4

Supplementary Figure5

Supplementary Figure6

Supplementary Figure7

Supplementary Figure8

Supplementary Figure9

Supplementary Figure10

Supplementary Figure11

Supplementary Figure12

## Data Availability

The ATAC-seq data of Japanese human melanoma samples were deposited to DRA013434, and RNAseq data were part of the sequencing data deposited to DRA011722 in the DNA Data Bank of Japan (DDBJ).
