# Supplementary material for: GREB1 isoform 4 is specifically transcribed by MITF and required for melanoma proliferation
Source: Oncogene. 2023 Sep 1;42(42):3142–56. doi: 10.1038/s41388-023-02803-6 (PMC10575781; doi:10.1038/s41388-023-02803-6)
Supplement: Supplementary file 1 — Supplementary Information [file 41388_2023_2803_MOESM1_ESM.docx]

**Supplementary Information**

**GREB1 isoform 4 is specifically transcribed by MITF and required for melanoma proliferation**

**Koei Shinzawa^1^*, Shinji Matsumoto^1,2^, Ryota Sada^1,2^, Akikazu Harada^1,2^, Kaori Saitoh^3^, Keiko Kato^3^, Satsuki Ikeda^3^, Akiyoshi Hirayama^3^, Kazunori Yokoi^4^, Atsushi Tanemura^4^, Keisuke Nimura^5,6^, Masahito Ikawa^7^, Tomoyoshi Soga^3^, and Akira Kikuchi^1,8^***

^1^Department of Molecular Biology and Biochemistry, Graduate School of Medicine, Osaka University, Suita, Osaka, Japan. ^2^Integrated Frontier Research for Medical Science Division, Institute for Open and Transdisciplinary Research Initiatives (OTRI), Osaka University, Suita, Osaka, Japan. ^3^Institute for Advanced Biosciences, Keio University, Tsuruoka, Yamagata, Japan. ^4^Department of Dermatology, ^5^Department of Genome Biology, Graduate School of Medicine, Osaka University, Suita, Osaka, Japan. ^6^Gunma University Initiative for Advanced Research, Gunma University, Maebashi, Gunma, Japan. ^7^Research Institute for Microbial Diseases, ^8^Center for Infectious Disease Education and Research, Osaka University, Suita, Osaka, Japan.

**Text summary**

Supplementary information includes the following contents

Supplementary Material and Methods

Supplementary References

Supplementary Tables S1-S7.

Supplementary Figures S1-S12 Legends.

**Supplementary Materials and Methods**

**Materials and chemicals.**

U2OS was purchased from the American Type Culture Collection (ATCC, Manassas, VA, USA). B16BL6 was from RIKEN BioResource Center (Ibaraki, Japan). Yummer1.7 was from Sigma-Aldrich (Steinheim, Germany). HeLa and B16F10 were from the Japanese Collection of Research Bioresources (JCRB, Osaka, Japan). U2OS and B16F10 cells were grown in Dulbecco’s modified Eagle’s medium (DMEM) supplemented with 10% FBS. HeLa and B16 BL6 cells were grown in RPMI1640 supplemented with 10% FBS. Yummer1.7 cells were grown in DMEM/F12 medium supplemented with 10% FBS and 1x non-essential amino acids. The cell lines were tested monthly for mycoplasma using the LookOut Mycoplasma PCR Detection Kit (Sigma Aldrich, MP0035). Cell line identity was confirmed using STR profiling. A771726 (Teriflunomide) was from Sigma-Aldrich (#SML0936).

The following antibodies were used: anti-GREB1 (#1) (#65171, 1:1000 for WB) and anti-GREB1 (#2) (#MABS62, 1:1000 for WB, 1:100 for IHC or IF) and anti-GREB1 (#3) (#28699-1-AP, 2.5 μg for IP) antibodies from Cell Signaling Technology (Beverly, MA, USA), Merck Millipore (Billerica, MA, USA), and Proteintech (Rosemont, IL, USA), respectively. Anti-GREB1 antibody (#1) is produced by immunizing rabbits with a synthetic peptide corresponding to residues surrounding Pro242 of human GREB1 protein, which was experimentally confirmed by recognizing the N-terminal half of human GREB1 protein. Anti-GREB1 antibody (#2) detected the C-terminal half of GREB1 corresponding to GREB1 Is4, especially the fragment A (amino acid Met1004 to Val1271). Rabbit polyclonal anti-GREB1 antibody (#3) was prepared with amino acids 1075-1212 as an immune antigen.

Anti-HSP90 (#610419, 1:1000 for WB) and Anti-clathrin (#610499, 1:1000 for WB) were from BD Biosciences (San Jose, CA, USA). anti-MITF (#2151, 1:1000 for WB), anti-GAPDH (#2118, 1:200 for IF), anti-CAD (#11933, 1:1000 for WB), anti-Estrogen Receptor α (D8H8) Ab (#8644, 1:1000 for WB), and anti-HA tag (1:200 for IHC) antibodies were from Cell Signaling Technology (Beverly, MA, USA). Anti-FLAG M2 Affinity Gel, anti-FLAG (#014-22383, 1:1000 for WB), anti-myc tag (#M192-3, 1:1000 for WB), anti-MITF (#HPA003259, 1:200 for IHC), and anti-SMAD2/3(#ab207447, for IP) antibodies were from Sigma-Aldrich, FUJIFILM WAKO (Osaka, Japan), MBL (Tokyo, Japan), ATLAS ANTIBODIES (Bromma, Sweden), and Abcam (Cambridge, UK), respectively.

**Plasmid construction and generation of stable lentiviruses**

Standard recombinant DNA techniques were used to design the plasmids harboring MITF(E318K), tagged GREB1 Is4 or tagged CAD and its various mutants. Mammalian expression vectors were constructed by subcloning target genes into the pcDNA3 vector. Lentiviral vectors were constructed by subcloning target genes into vector CSII-CMV-MCS-IRES2-Bsd or CSIV-RfA-TRE-EF-KT for Dox-inducible expression, kindly provided by Dr. H. Miyoshi (RIKEN BioResource Center, Ibaraki, Japan). Lentiviruses were generated by transfecting the lentiviral vectors and the packaging vectors (pCAG-HIV-gp and pCMV-VSV-G-RSV-Rev) into 293T cells using Lipofectamine2000 transfection reagent (Life Technologies/Thermo Fisher Scientific).

**Immunofluorescence staining**

Cells (2.11 x 10^4^ cells/cm^2^) grown on glass coverslips were fixed for 10 min at room temperature in PBS containing 4% (w/v) paraformaldehyde and permeabilized in PBS containing 0.2% (w/v) Triton X-100 and 5% (w/v) Skim milk for 1 h. The cells were then incubated with primary antibodies overnight at 4°C and secondary antibodies conjugated with fluorescence dye for 1 h at room temperature. Target protein localization was visualized using an LSM880 laser confocal microscope (Carl-Zeiss, Jena, Germany).

**Open source data analysis.**

Cancer cell line data were obtained from the Gene Expression database of Normal and Tumor tissues 2 (GENT2) (<http://gent2.appex.kr/gent2/)>. The *R* value used in Widmer plot for proliferative and invasive correlation coefficients was derived from HOPP data (<http://jurmo.ch/hopp/hopp_default_data.php>). Transcribed *GREB1* genome locations in TCGA datasets were obtained using TCGA Splicing Variants DB (http://www.tsvdb.com/instruction.html). DepMap data were obtained from Cancer Dependency Map (<https://depmap.org/portal/>). Clinical TCGA data for tumor patients were obtained from the UCSC Xena site (http://xena.ucsc.edu). Mouse *greb1* and human *GREB1* isoforms data was derived from ensemble data (https://asia.ensembl.org/index.html). *GREB1* expression data in human normal tissue were obtained from GTEx portal site (https://gtexportal.org/home/). The *greb1* genome insertion sites of the SB transposon were derived from SBCDDB (Sleeping Beauty Cancer Driver Database) site (https://sbcddb.moffitt.org).

**Generation of Colo679 MITF- and GREB1 Is4-KO cells**

KO cells were generated using CRISPR/Cas9 technology. The gRNA sequences for human *MITF* were 5′-GTCCGAATCGGGGATCGTGC-3′ and 5′-ACAACTCTCGATCTCACGGA-3′ and those　for human *GREB1 Is4* were 5′-TCAGTGGGATGGACCCGCAT-3′ and 5′-GTTCAACCGCTTCAGCGTGA-3′. The gRNA was cloned into the BbsI site of the pX330 vector expressing hCas9 and the blasticidin resistance gene (addgene 42230). The pX330-GREB1 Is4 and pX330-MITF targeting plasmids were transfected separately into Colo679 cells using Lipofectamine 2000 reagent (Life Technologies/Thermo Fisher Scientific). The transfected cells were selected with 5 μg/mL blasticidin S for two days. Single colonies were picked, and MITF-KO and GREB1 Is4-KO clones were identified by WB.

**ChIP assay**

ChIP was performed using the SimpleChIP® Enzymatic Chromatin IP Kit (Cell Signaling Technology, Beverly, MA, USA) according to the manufacturer’s instructions, except for changes in the magnetic beads. Cross-linked chromatin was broken into 200–1000 bp fragments by enzymatic digestion. The chromatin was immunoprecipitated overnight with 2 μg of antibody at 4°C with rotation. SureBead Protein G magnetic beads (Bio-Rad, Hercules CA, USA) (100 μl) were added to each sample. The samples were then incubated at 4°C for 2 h with rotation. The immunoprecipitated chromatin was washed three times with a low-salt solution and once with a high-salt solution. Finally, the immunoprecipitated chromatin was purified and analyzed by qRT-PCR. The following primers were used for PCR analysis:

GREB1 Is4 promoter (intron18-19) region 5‘-CAAATGAGGTCTTTGTGTGTGG-3‘, 5’-AAGATCATTCTCGTAGCACTGG-3‘;

GREB1 negative control (exon24) region 5’-CCCTACCCAGAGACAGACCA-3’, 5’-CAGGAGGTGAGTTCACAGCA-3’.

PMEL promoter (intron1-2) region 5‘-GGACATTCCAGACCCTCTCA-3‘, 5’-ATGGGTACGGGTGAACACAG-3‘

**Reporter gene assay**

Putative GREB1 Is4 promoter (-82-0) or (-315-0) including MITF-binding E-box was cloned into BglII site of pGL4 vector. 293T cells were transfected with FLAG-HA-WT MITF in pcDNA3, GREB1 Is4 promoter in pGL4-Luc, and pCMV-LacZ. At 24 h after transfection, the cells were lysed, and the luciferase activity was measured with PicaGene reagent (Toyo Ink, Tokyo, Japan) as described previously [1]. β-Galactosidase activities were determined to normalize the transfection efficiency.

**Single-cell RNA-seq data analysis (scRNA-seq)**

scRNA-seq data of nine patient-derived cultures and A375 cell lines was downloaded from Scope (https://scope.aertslab.org/#/Wouters_Human_Melanoma/*/welcome) [2]. Data in loom file contains already calculated tSNE and annotations of cell states for each cell. scRNA-seq data of patient-derived xenograft melanoma cells isolated at different time points of treatment was downloaded from GEO accession number GSE116237 [3]. Gene expression matrix was used for analysis. As a quality control, genes that are detected in less than 3 cells were filtered out. Then pre-processing was conducted by sc.pp.normalize_total (target_sum=1e4) and sc.pp.log1p implemented in Scanpy. Scanpy v1.6.0 and python 3.6.13 were used to analyze and visualize data.

**Immunohistochemical analysis**

Immunohistochemical (IHC) staining was performed on tissue sections using the Dako Real™ EnVision™ Detection System (DAKO, Carpentaria, CA, USA), according to the manufacturer’s recommendations. Antigen retrieval was performed with Target Retrieval Solution (DAKO). Endogenous peroxidase activity was blocked with Peroxidase Blocking Solution (DAKO), and the sections were then incubated with Blocking One Histo (Nacalai tesque, Kyoto, Japan) to block nonspecific antibody-binding sites. Tissue specimens were incubated with anti-GREB1 (1:100) (#2) or anti-MITF (1:100) antibody for 16 h at 4°C, and the target proteins were detected by subsequent incubation with secondary antibodies conjugated with alkaline phosphatase for 1 h. Staining was visualized with Warp Red (Biocare Medical, Concord, CA, USA) as the chromogen. The sections were counterstained with 0.01% (w/v) hematoxylin.

IHC scores for MITF and GREB1 protein expression (0, negative; 1, weak; 2, intermediate; 3, strong) were determined by considering both the staining intensity and staining area in the tumor lesions by three and two independent investigators, respectively. When there were discrepancies in an IHC score, all investigators re-scored the section to reach a consensus.

The optical density of individual cells for GREB1 and MITF staining is quantified and pseudo-colored and the localization was mapped by Halo software (Indica Labs Inc., Albuquerque, NM, USA).

**Cell proliferation assay**

Cells were seeded at a density of 2 × 10^3^ cells/100 μl in 96 well plates. siRNAs or ASOs (10 nM) were transfected with RNAiMax (Life Technologies/Thermo Fisher Scientific). Cell proliferation was measured using CyQUANT NF (Life Technologies/Thermo Fisher Scientific), according to the manufacturer’s instructions. The fluorescence intensity reflects the cell number in this assay.

For the soft agar assays, 2 × 10^4^ cells/1.5 ml of DMEM containing 10% FBS and 0.375% agar were seeded on a 2.5 ml layer of 0.75% agar in 6-well plates. Growth medium (1 ml) was added and refreshed every three days. Two weeks after plating, colonies were imaged, and their numbers and areas were quantified using ImageJ software.

**Cell death and senescence assay**

Cells detached with collagenase/EDTA and culture medium were combined and centrifuged to collect cells. Pelleted cells were washed twice with PBS and stained with 2 μg/ml PI and 1 μg/ml Hoechst33342. PI staining was visualized using an LSM880 laser confocal microscope (Carl-Zeiss, Jena, Germany). Cell senescence was measured using Senescence β-galactosidase staining kit (Cell Signaling Technology), according to the manufacturer’s instructions.

**Generation of ASOs targeting for GREB1**

Phosphorothioate 15-mer ASOs containing AmNA monomers were synthesized by Gene Design (Ibaraki, Japan) [4]. The sequences of the ASOs are as follows: Control ASO, T(Y)^a^g^A(Y)^g^a^G(Y)^t^a^5(Y)^c^c^A(Y)^t^c; GREB1 ASO #6434, 5(Y)^G(Y)^A(Y)^a^t^g^g^c^a^g^g^a^5(Y)^A(Y)^g; GREB1 ASO #7724, A(Y)^T(Y)^T(Y)^g^a^g^g^g^t^a^g^g^5(Y)^A(Y)^a. The abbreviations used in the sequences are as follows: N(Y), AmNA; 5(Y), AmNA_mC; ^, Phosphorothioated.

**Xenograft melanoma tumor formation assay and ASO treatment**

Colo679 cells (2.5 × 10^6^ cells) were resuspended in 150 μL of 50 % high concentration Matrigel (Corning, Glendale, AZ, USA) and inoculated subcutaneously into the dorsal flank of 8-week-old male BALB/cAJcl nu/nu mice (nude mice; CLEA Japan). Beginning three weeks post-inoculation, control ASO (n = 6) or GREB1 ASO-7724 (100 μg/body [~5.0 mg/kg]; N = 6/group) was administered subcutaneously every three days. Three weeks after inoculation, mice were euthanized. IVIS images of the subcutaneous tumors were collected using the IVIS Lumina system (Xenogen, Alameda, CA, USA), and the dissected tumors were weighed.

**Generation of the GREB1 Is4 Tg Mouse Model**

The targeting vectors were cloned by modifying the pCAGGS vector containing a CAG promoter. Human FLAG-HA-tagged *GREB1 Is4* preceded by a loxP-flanked 3 x STOP cassette was inserted downstream of the CAG promoter. The fragment was amplified by PCR and subcloned between the mouse *rosa 26* intron1 left (207 bp) and right (194 bp) arms of the pLSODN-1 vector to generate the final targeting construct. Embryonic stem (ES) cells were electroporated with Cas9 mRNA, rosa 26 sgRNA and the targeting vector, and ES cell clones containing the targeting construct were identified by PCR. The clones were microinjected into C57BL/6N blastocysts to generate chimeric mice that exhibited germline transmission of the targeting construct allele. The genotypes of mice were assessed by PCR using the following primers:

*Tyr-CreER*, 5’- CCC ACA TCA GGC ACATGA GT-3’,

5’-AGTTCCCACACTTAACAGCCCCATCCTCTC-3’ and

5’-AGGAAGCCCTGGACGTGTGGAGGGATCGTG-3’;

*BRAF^V600E^*

5’-TGTGCCCTATAAAGTGAGTATTTTTGTGGCAACTGC-3’ and

5’-GGCCCAGATACACTTTAACTCTGCTGGGAAAGCGGC-3’;

*PTEN^flox^*

5’-CTCCCACCAATGAACAAACAGT-3’ and

5’-GTGAAAGTGCCCCAACATAAGG-3’;

*GREB1 Is4*

5’-AACGTGCAGTACAACCAGAACCGGTTCCTG-3’ and

5’-TACAGCAGCAGCCCACTGAACAGCAAGTC-3’;

*rosa26*

5’-CACGTTTCCGACTTGAGTTGCCTCAAGAGG-3’ and

5’-CTCGGGTGAGCATGTCTTTAATCTACCTCG-3’.

**Isolation of GREB1 Is4-interacting proteins.**

Colo679, HeLa, or U2OS cells stably expressing FLAG-HA-GREB1 Is4 (5 × 10^6^ cells) were seeded in three 10-cm dishes and washed three times with 10 ml of PBS. Each plate of cells was lysed with 0.7 ml NP-40 buffer (20 mM Tris-HCl [pH 8.0][10% glycerol, 137 mM NaCl, and 1% NP-40) containing protease and phosphatase inhibitors. After centrifugation for 7 min, the supernatant was incubated with 100 μl of a 50% slurry of M2 agarose beads for 1 h at 4°C. The beads were washed three times with 1 ml of NP-40 buffer and then incubated twice with FLAG peptide (0.5 mg/mL) in 50 μl of NP-40 buffer for 30 min at 4°C to elute proteins. The GREB1 Is4–interacting proteins were detected by silver staining. The candidate bands were cut from the gel, and the proteins were identified by trypsin digestion followed by liquid chromatography (LC)- mass spectrometry (MS)/MS analysis.

**Immunoprecipitation.**

Cells in 100-mm diameter dish were lysed in 1 ml of lysis buffer (10mM Tris-HCl [pH 7.4], 140 mM NaCl, 5 mM EDTA, 10 % glycerol, 1% NP40, 25 μM NaF, 20 μg/ml leupeptin, 20 μg/ml aprotinin, and 10 μM PMSF). After centrifugation, lysates were immunoprecipitated with indicated antibodies conjugated with Sure protein G magnetic beads (Biorad). After washing with lysis buffer three times, immunoprecipitates were probed with indicated Abs.

**Proximity ligation assay (PLA)**

PLA was performed according to the manufacturer's protocol (Sigma) as described previously [5]. Briefly, colo679 cells grown on glass coverslips were fixed and permeabilized in PBS containing 0.2% (w/v) Triton X-100 and 2 mg/ml BSA for 10 min. The glass coverslips were blocked and incubated with anti-GREB1 mouse antibody (#2) and anti-CAD rabbit antibody diluted in blocking buffer for 1 h at RT. After washing, the coverslips were incubated with Duolink PLA anti- mouse minus and PLA anti-rabbit plus proximity probes. PLA dots were counted using an LSM880 laser confocal microscope.

***De novo* pyrimidine synthesis assay (Flux analysis)**

Colo679 cells (3 × 10^6^ cells) were incubated in DMEM medium for 15 h in 10-cm dishes, washed with serum- and glutamine-free DMEM, and then labeled with 4 mM ^15^N^13^C-glutamine in the presence of 4 mM cold glutamine for 60 min. The cells were washed twice with 10 ml of 5% mannitol and cell extracts were prepared by incubating the cells for 10 min in 1 ml of MeOH containing three internal standards (5 μM L-Methionine sulfone, MES, and CSA). The extracts were collected into a 1.5 mL microcentrifuge tube and lyophilized. The samples from five dishes were pooled into one tube, and the intracellular concentrations of metabolites were analyzed by CE-MS, ion chromatography-mass spectrometry (IC-MS) and liquid chromatography mass spectrometry (LC-MS).

CE-MS experiments were performed using Agilent 7100 CE capillary electrophoresis (Agilent Technologies, Waldbronn, Germany), the Agilent 6230 LC/MSD TOF system (Agilent Technologies, Palo Alto, CA, USA), an Agilent1100 series binary HPLC pump, and the G1603A Agilent CE-MS adapter- and G1607A Agilent CE-ESI-MS sprayer kit. For anionic metabolite analysis, the original Agilent stainless ESI needle was replaced with the Agilent G7100-60041 platinum ESI needle 53. System control and data acquisition were performed using an Agilent MassHunter Workstation, and data analysis was performed with Keio MasterHands software.

IC-MS was performed using a Dionex ICS-5000^+^ system connected to a Q Exactive Orbitrap MS system (Thermo Fisher Scientific, San Jose, CA, USA). Sample injection was performed with a Dionex WPS-3000TBPL autosampler, and an Agilent 1100 series capillary HPLC pump was used to deliver the make-up solution. LC-MS was performed using an Agilent 1290 Infinity LC system (Agilent Technologies, Santa Clara, CA, USA) equipped with a Q Exactive Orbitrap MS system.

**CE-MS method for anionic metabolite analysis.**

A commercially available COSMO(+) capillary (chemically coated with a cationic polymer) (50 μm i.d. × 105 cm total length) (Nacalai Tesque, Kyoto, Japan) was used with a 50 mM ammonium acetate solution (pH 8.5) as the electrolyte. Samples (30 nL) were injected at 50 mbar for 30 sec, and a -30 kV voltage was applied. Ammonium acetate (5 mM) in 50% methanol-water (v/v) containing 0.1 μM Hexakis (2,2-difluoroethoxy) phosphazene was delivered as the sheath liquid at 10 μL/min. ESI-TOFMS was conducted in the negative ion mode; the capillary voltage was set at 3,500 V. For TOFMS, the fragmentor-, skimmer-, and Oct RFV voltage were set at 100 V, 50 V, and 200 V, respectively. Automatic recalibration of each acquired spectrum was performed using the reference masses of the reference standards, i.e., the ^13^C isotopic ion of the deprotonated acetic acid dimer ([2CH3COOH-H]-, m/z 120.0384), and Hexakis + deprotonated acetic acid (m/z 680.03554) provided the lock mass for exact mass measurements.

**LC-MS method for cationic metabolite analysis.**

Separation was performed on a HILIC-Z column (150 × 2.1 mm, 2.7 μm; Agilent Technologies). The injection volume was 1 μL, and the column temperature was maintained at 40°C. The mobile phase consisted of 20 mM ammonium formate + 0.25% (v/v) formate (A) and 20 mM ammonium formate + 0.25% formate in 90% (v/v) acetonitrile (B). The mobile phase flow rate was 0.25 mL/min, and the gradient condition was as follows: 0–15 min, 100% to 70% B; 15–20 min, 70% to 10% B; 20–23 min, 10% B; and 23–30 min, 100% B. The Q Exactive mass spectrometer was operated in a heated ESI positive-ion mode using the following source parameters: auxiliary gas temperature = 300°C, auxiliary gas flow rate = 10 (arbitrary units), spray voltage = 3.5 kV, capillary temperature = 250°C, sheath gas flow rate = 40 (arbitrary units), and S-lens = 35 (arbitrary units). Data were acquired in full MS scan mode and the parameters were as follows: resolution, 35,000; auto gain control target, 3 × 10^6^; maximum ion injection time, 200 ms; scan range, 50–750 *m/z*.

**IC-MS method for anionic metabolite analysis**

The anionic metabolites were separated using a Dionex IonPac AS11-HC-4 μm column (250 × 0.4 mm, 4 μm; Thermo Fisher Scientific) [6]. The column temperature was maintained at 35°C. The eluent flow rate was 20 μL/min, and the following concentration gradient of potassium hydroxide was used: 1 mM from 0 to 2 min, 20 mM at 16 min, 100 mM at 35 min, held at this concentration until 40 min, then decreased to the initial concentration within 0.1 min and held for 5 min (total analysis time was 45.1 min). Isopropanol containing 0.1 % acetic acid was delivered as the make-up solution at 5 μl/min to enhance the ionization efficiency. The mass spectrometer was operated in the ESI negative-ion mode. The ESI parameters were as follows: sheath gas, 20 (arbitrary units); auxiliary gas, 10 (arbitrary units); sweep gas, 0; spray voltage, 4.0 kV; capillary temperature, 300°C; S-lens, 50 (arbitrary units). Data were acquired in full MS scan mode with the following parameters: resolution, 140,000; auto-gain control target, 3 × 10^6^; maximum ion injection time, 100 ms; scan range, 70–1,000 *m/z*.

***In Vitro* CPSase assay**

The CPSase assay was performed as previously described [7]. Briefly, the lysates from 293T cells expressing FLAG-HA-GREB1 Is4 or FLAG-HA-CAD were immunoprecipitated with M2 agarose beads, and the FLAG-HA-tagged proteins were eluted using a FLAG peptide. The purified proteins were resolved by SDS-PAGE followed by Coomassie Brilliant Blue (CBB) staining. The protein concentration was determined using BSA as a standard. FLAG-HA-GREB1 Is4 (0.5 μg) and FLAG-HA-CAD (0.5 μg) were incubated in a 1 -mL reaction mixture containing 100 mM Tris-HCl (pH 8.0), 100 mM KCl, 3 mM ATP, 1 mM DTT, 7.5% dimethyl sulfoxide, 10% glycerol, 5 mM MgCl_2,_ 3.3 mM glutamine, 17.5 mM aspartate, and 5 mM ^14^C-labeled sodium bicarbonate (37 kBq) (1.48–2.22 GBq/mmol, PerkinElmer Life Sciences) for 60 min at 37°C. When necessary, 0.5 mM UTP or 0.2 mM PRPP was added. The reaction was quenched by adding trichloroacetic acid (TCA) at a final concentration of 20%. The samples were heated for 1 h at 95°C, and then powdered dry ice was added to the tubes to eliminate excess CO_2_. The ^14^C-labeled metabolites were counted with a liquid scintillation counter.

***De novo* RNA synthesis assay**

Colo679 cells (3 × 10^5^) were treated with control or GREB1 siRNA for 48 h, and then all culture medium was replaced by medium containing 4.3 mM ^14^C-labeled sodium bicarbonate (74 kBq) [8]. Cells were incubated for the indicated times, and total RNA was extracted. The radioactivity in the total RNA was determined using a liquid scintillation counter.

**CellTiter-Glo assay.**

Melanoma cell lines were plated at 1.0 x 10^4^ cells/well containing indicated concentration of A771726 (Sigma-Aldrich) in triplicate 96 wells. Cell viability was measured at day 4 using CellTiter-Glo assay kit (Promega). All values are expressed as the % of luciferase relative to the DMSO control.

**Supplementary References**

1 Yamamoto, H, Ihara, M, Matsuura, Y, Kikuchi, A. Sumoylation is involved in beta-catenin-dependent activation of Tcf-4. EMBO J. 2003; 22: 2047-2059.

2 Wouters, J, Kalender-Atak, Z, Minnoye, L, Spanier, KI, De Waegeneer, M, Bravo Gonzalez-Blas, C *et al.* Robust gene expression programs underlie recurrent cell states and phenotype switching in melanoma. Nat Cell Biol. 2020; 22: 986-998.

3 Rambow, F, Rogiers, A, Marin-Bejar, O, Aibar, S, Femel, J, Dewaele, M *et al.* Toward

Minimal Residual Disease-Directed Therapy in Melanoma. Cell. 2018; 174: 843-855 e819.

4 Matsumoto, S, Yamamichi, T, Shinzawa, K, Kasahara, Y, Nojima, S, Kodama, T *et al.* GREB1 induced by Wnt signaling promotes development of hepatoblastoma by suppressing TGFβ signaling. Nat Commun. 2019; 10: 3882.

5 Osugi, Y, Fumoto, K, Kikuchi, A. CKAP4 regulates cell migration via the interaction with and recycling of integrin. Mol Cell Biol. 2019; 39: e00073-00019.

6 Hirayama, A, Tabata, S, Kudo, R, Hasebe, M, Suzuki, K, Tomita, M *et al.* The use of a double coaxial electrospray ionization sprayer improves the peak resolutions of anionic metabolites in capillary ion chromatography-mass spectrometry. J Chromatogr A. 2020; 1619: 460914.

7 Sato, T, Akasu, H, Shimono, W, Matsu, C, Fujiwara, Y, Shibagaki, Y *et al.* Rheb protein binds CAD (carbamoyl-phosphate synthetase 2, aspartate transcarbamoylase, and dihydroorotase) protein in a GTP- and effector domain-dependent manner and influences its cellular localization and carbamoyl-phosphate synthetase (CPSase) activity. J Biol Chem. 2015; 290: 1096-1105.

8 Sigoillot, FD, Berkowski, JA, Sigoillot, SM, Kotsis, DH, Guy, HI. Cell cycle-dependent regulation of pyrimidine biosynthesis. J Biol Chem. 2003; 278: 3403-3409.

**Table S1 The list of top 25 genes positively correlated with *GREB1* expression in SKCM TCGA dataset**

| *GENE* name | *R* value | *P* value |
| --- | --- | --- |
| ***TBC1D16*** | 0.609 | 2.1E-35 |
| ***PPM1H*** | 0.606 | 3.9E-35 |
| *TRIM63* | 0.605 | 4.4E-35 |
| ***GPR143*** | 0.596 | 7.6E-34 |
| ***PMEL*** | 0.57 | 3.3E-30 |
| ***SLC45A2*** | 0.57 | 2.9E-30 |
| *TSPAN10* | 0.559 | 9.5E-29 |
| *TTYH2* | 0.557 | 1.2E-28 |
| ***MITF*** | 0.556 | 1.8E-28 |
| *ESRP1* | 0.553 | 3.7E-28 |
| ***MLANA*** | 0.538 | 2.5E-26 |
| *FAM69B* | 0.535 | 5.8E-26 |
| ***SEMA6A*** | 0.533 | 1.1E-25 |
| ***TRPM1*** | 0.532 | 1.2E-25 |
| ***VAT1*** | 0.53 | 1.9E-25 |
| *CABLES1* | 0.527 | 3.9E-25 |
| ***BIRC7*** | 0.521 | 1.8E-24 |
| ***HPS4*** | 0.511 | 2.8E-23 |
| *SLC25A13* | 0.51 | 3.1E-23 |
| ***CLCN7*** | 0.504 | 1.5E-22 |
| ***TYR*** | 0.501 | 2.7E-22 |
| ***ASAH1*** | 0.5 | 3.6E-22 |
| *ZNF703* | 0.5 | 3.3E-22 |
| ***PIR*** | 0.499 | 4.3E-22 |

Pearson’s correlation coefficient between *GREB1* expression in melanoma TCGA dataset (N=470) were investigated and shown in order of high *R* value. The gene names that have already been reported as MITF target genes are shown in bold.

**Table S2 Univariate analysis of overall survival of malignant melanoma cases by Cox’s proportional hazard model**

| Parameters | Number | Hazard ratio | 95% CI | *P* value |
| --- | --- | --- | --- | --- |
| Sex (male/female) | 53/36 | 0.514 | 0.256-1.029 | 0.0583 |
| Age (≥65years/<65years) | 34/55 | 1.967 | 0.967-4.223 | 0.0694 |
| pStage ( III, IV / 0-II ) | 57/32 | 3.118 | 1.488-6.639 | 0.0026 |
| MITF expression (high / low) | 34/55 | 2.023 | 1.012-4.120 | 0.0475 |
| GREB1 expression (high / low) | 30/59 | 1.050 | 0.507-2.119 | 0.8931 |
| ICI /MMT administration  (non-treated/treated) | 71/18 | 1.496 | 0.609-3.581 | 0.3675 |

Tis: tumor in situ, T1: tumor thickness is less than 1 mm, T2: tumor thickness is more than 1.01 mm but less than 2.00 mm, T3: tumor thickness is more than 2.01 mm, but less than 4 mm, T4: tumor thickness is more than 4.0 mm, N0: No metastatic Nodes, N1: there is evidence of involvement of one regional lymph node, N2: there is evidence of involvement of 2 to 3 regional lymph nodes, N3: >4 metastatic nodes, or matted nodes, or in transit metastases/satellites with metastatic nodes, M1: there is evidence of distant metastasis, CSD: chronic sun-induced damage, ICI/MTT: Immune checkpoint inhibitors/molecular targeted therapeutics.

**Table S3 The list of GREB1 Is4 binding proteins in Colo679 cells**

| Band  number | Gene  name | Molecular  Weight (kDa) |  | Band  number | Gene  name | Molecular  weight (kDa) |
| --- | --- | --- | --- | --- | --- | --- |
| 1 | *PRKDC* | 469 |  | 8 | *MTR* | 141 |
| 1 | *PLEC* | 532 |  | 8 | *INPP5F* | 128 |
| 1-12 | *GREB1* | 216 |  | 8 | *SMC2L1* | 136 |
| 2 | *SPTBN1* | 275 |  | 8 | *SF3B3* | 136 |
| 3 | *FASN* | 273 |  | 9 | *MYH9* | 227 |
| 3 | ***CAD*** | 236 |  | 9 | *HSP90AA1* | 85 |
| 3 | *PRIC295* | 293 |  | 9 | *IMMT* | 79 |
| 4 | *ASCC3* | 251 |  | 9 | *DNAJC10* | 91 |
| 4 | *CKAP5* | 226 |  | 9 | *HSP90AB1* | 83 |
| 5 | *Ubiquitin C variant (Fragment)* | 147 |  | 9-10 | *HSPA8* | 71 |
| 5 | *VWA8* | 215 |  | 10 | *HSPA5* | 72 |
| 5 | *POLR1A* | 195 |  | 10 | *HSPD1* | 61 |
| 5 | *hCG_1991735* | 189 |  | 10 | *LMNA* | 74 |
| 5 | *ARFGEF2* | 202 |  | 10 | *HSPA1B* | 70 |
| 5 | *PARP4* | 193 |  | 10 | *DDX3X* | 81 |
| 6 | *EPRS* | 171 |  | 11 | *SLC25A5* | 33 |
| 6 | *CLTC* | 192 |  | 11 | *SLC25A6* | 33 |
| 6 | *UGGT1* | 177 |  | 11 | *YWHAZ* | 28 |
| 6 | *EIF3A* | 167 |  | 11 | *PHB* | 30 |
| 7 | *BAG6* | 119 |  | 11 | *RPL7* | 30 |
| 7 | *HERC2* | 527 |  | 11 | *SLC25A3* | 40 |
| 8 | *LRPPRC* | 158 |  | 12 | *UBR5* | 309 |
| 8 | ELP1 | 150 |  | 12 | *NES* | 177 |

The silver-stained bands shown in Fig. 6B was excised and the protein was identified by trypsin digestion followed by LC-MS/MS analysis. All the identified proteins with more than 6 peptides are shown. *CAD* gene is shown in bold.

**Table S4 The list of GREB1 Is4 binding proteins in Hela cells**

| Band number | Gene name | Molecular weight (kDa) |
| --- | --- | --- |
| 1 | *HUWE1* | 481 |
| 2 | *PRKDC* | 469 |
| 3 | ***CAD*** | 236 |
| 4 | *HRNR* | 282 |
| 4 | *DSP* | 332 |
| 4 | *BCLAF1* | 106 |
| 4 | *JUP* | 82 |
| 4 | *CPS1* | 165 |
| 4 | *OTUD4* | 124 |
| 4 | *BAG6* | 119 |
| 5 | *HNRNPU* | 81 |
| 5-8 | *GREB1* | 216 |
| 6 | *HSP90AB1* | 83 |
| 6 | *HSP90AA1* | 85 |
| 6 | *DHX15* | 91 |
| 7 | *Tubulin alpha chain* | 46 |
| 7 | *TUBB* | 50 |
| 7 | *HNRNPH1* | 51 |
| 7 | *ATP5F1A* | 60 |
| 7 | *RUVBL1* | 50 |
| 8 | *EEF1A1* | 50 |
| 8 | *EEF1G* | 50 |
| 8 | *RPL3* | 46 |
| 8 | *DNAJA1* | 45 |
| 9 | *SLC25A6* | 33 |
| 9 | *RPS4X* | 30 |
| 10 | *BAG2* | 24 |
| 10 | *RPL9* | 25 |
| 10 | *PSMA2* | 26 |

The silver-stained bands shown in Supplementary Fig. S9A was excised and the protein was identified by trypsin digestion followed by LC-MS/MS analysis. All the identified proteins with more than 6 peptides are shown. *CAD* gene is shown in bold.

**Table S5 The list of GREB1 Is4 binding proteins in U2OS cells**

| Band number | Gene name | Molecular weight (kDa) |
| --- | --- | --- |
| 1 | ***CAD*** | 236 |
| 2 | *cDNA FLJ76863* | 63 |
| 2 | *CKAP4* | 66 |
| 3 | *cDNA FLJ31447* | 53 |
| 4 | *GREB1* | 216 |
| 4 | *cDNA FLJ31447* | 53 |
| 4 | *T-complex protein 1 subunit delta* | 58 |
| 4 | *Chaperonin containing TCP1* | 59 |
| 4-6 | *STK38* | 54 |
| 5 | *CCT2* | 57 |
| 6 | *TUBB* | 50 |
| 6 | *GREB1* | 216 |
| 6 | *Tubulin alpha chain* | 46 |
| 6 | *HNRNPH1* | 51 |
| 7 | *RPL3* | 46 |
| 7 | *DNAJA1* | 45 |
| 8 | *SLC25A5* | 33 |
| 8 | *RPS4X* | 30 |
| 10 | *cDNA FLJ13673* | 24 |
| 10 | *RPL15* | 24 |
| 10 | *PSMA2* | 26 |

The silver-stained bands shown in Supplementary Fig. S9A was excised and the protein was identified by trypsin digestion followed by LC-MS/MS analysis. All the identified proteins with more than 6 peptides are shown. *CAD* gene is shown in bold.

**Table S6 The Metabolite set enrichment analysis**

| Metabolite Set | Total | Hit | Expect | *P*-value | FDR |
| --- | --- | --- | --- | --- | --- |
| Pyrimidine metabolism | 39 | 5 | 0.512 | 9.95E-05 | 0.00836 |
| Arginine biosynthesis | 14 | 3 | 0.184 | 6.45E-04 | 0.0271 |
| Purine metabolism | 65 | 5 | 0.854 | 0.00 | 0.0321 |
| Alanine, aspartate and glutamate metabolism | 28 | 2 | 0.368 | 0.05 | 1.0 |
| Glycine, serine and threonine metabolism | 33 | 2 | 0.434 | 0.07 | 1.0 |
| Ascorbate and aldarate metabolism | 8 | 1 | 0.105 | 0.10 | 1.0 |
| Biotin metabolism | 10 | 1 | 0.131 | 0.12 | 1.0 |
| Aminoacyl-tRNA biosyntesis | 48 | 2 | 0.631 | 0.13 | 1.0 |
| Mannose type O-glycan biosynthesis | 17 | 1 | 0.223 | 0.20 | 1.0 |
| Pentose and glucuronate interconversions | 18 | 1 | 0.237 | 0.21 | 1.0 |

Metabolite set enrichment analysis was performed using the metabolome dataset from control or *GREB1* siRNA-treated cells (ratio<1.5, log2FC<-0.58, n=20). Pyrimidine metabolism metabolite set.

**Table S7 The list of primer sequences for qPCR**

| *Gene name* | Forward primer | Reverse primer |
| --- | --- | --- |
| *human GAPDH* | 5’-TCCTGCACCACCAACTGCTT-3’ | 5’-TGGCAGTGATGGCATGGAC-3’ |
| *human GREB1 Nter1* | 5’-GGTCCGGCTGTTTTCAACGGCAAAGATTCC-3’ | 5’-GCAGGACATGCCTGCGCTCTCATACTTAGC-3’ |
| *human GREB1 Nter2* | 5’-ATCTGGACCTGGGATCCTTTGAGAAGGTGG-3’ | 5’-GGACAAAAAGTGTATGCGGGTTCTGGAAGG-3’ |
| *human GREB1 Cter1* | 5’-CCAGGATGCTTGTTCGGCTCACAGAAGTGG-3’ | 5’-GGCTTCCGGGACATCCCTCTGTCTTCACTC-3’ |
| *human GREB1 Cter2* | 5’-AACTTCATCATCCTGAACGTGGACCTGACC-3’ | 5’-CCCACCACGATCTGCTTCTTCATCACGCTG-3’ |
| *human MITF* | 5’-CTCGAGCTCATGGACTTTCC-3’ | 5’-CCAGTTCCGAGGTTGTTGTT-3 |
| *human MALA* | 5’-GCTCATCGGCTGTTGGTATT-3’ | 5’-ATAAGCAGGTGGAGCATTGG-3’ |
| *human PMEL* | 5’-ATGTGCCTCTTGCTCATTCCAGCTC-3’ | 5’-CTGTCTCCAAAGTCCCAGGTGTAGG-3’ |
| *human GREB1 Is4* | 5’-TCCTTCCACATCACATCCAA-3’ | 5’-CAGTCGACAGACAGCACTGG-3’ |
| *human DCT* | 5’-CATGGTTCCTTTCTTCCCTCCAGTG-3’ | 5’-AGACCAACCAAAGCCACCAGTGTTC-3’ |
| *human CDK2* | 5’-CTCACTGGCATTCCTCTTCCCCTCATCAAG-3’ | 5’-AGTACGAACAGGGACTCCAAAAGCTCTGGC-3’ |
| *human MYC* | 5’-GTGGTCTTCCCCTACCCTCTCAACGACAGC-3’ | 5’-GTGATCCAGACTCTGACCTTTTGCCAGGAG-3’ |
| *human RPA1* | 5’-TGAAGATTGGCAATCCAGTGCCCTATAATG-3’ | 5’-ACCACTTTGGACTGTGTTCCCCCAGAAGTG-3’ |
| *human MCM5* | 5’-GCCTGGCTGCCCTCCCAAATGTCTATGAGG-3’ | 5’-TCTCCACAAACTTCAGAAGCTGGGACTTGG-3’ |
| *human PLK1* | 5’-CACCGGCGAAAGAGATCCCGGAGGTCCTAG-3’ | 5’-GTGGGCGAGGCTGCGGTGAATGGATATTTC-3’ |
| *human XRCC3* | 5’-CTCGGCACACACTGTGCCCCACAAAACTTC-3’ | 5’-GATACACCGGGACCCTCCTTCCTCTCAACC-3’ |
| *human LIG1* | 5’-TTTGTGGAGACAGAGGGCGAGTTTGTCTTC-3’ | 5’-TTCAGCTTGAGCCAGTTGTGCGATCTCTTG-3’ |
| *human CDKN1A* | 5′-ATGAAATTCACCCCCTTTCC-3’ | 5′-CCCTAGGCTGTGCTCACTTC-3′ |
| *human CDKN1B* | 5’-ACCCCTAGAGGGCAAGTACG-3’ | 5’-ATCAGTCTTTGGGTCCACCA-3’ |
| *human AXL* | 5’-CAAGGCCAGGACACCCCAGAGGTGCTAATG-3’ | 5’-CCAGGGCCACGAGAAGGCAGGAGTTGAAGG-3’ |
| *human ZEB1* | 5’-GAATGCACAACCAAGTGCAGAAGAGCTCTC-3’ | 5’-GGGTTCATTTGCATTTGCAGATTGAGGCTG-3’ |
| *human TGFB1* | 5’-GGGACTATCCACCTGCAAGA-3’ | 5’-CCTCCTTGGCGTAGTAGTCG-3’ |
| *human WNT5A* | 5’-CACGCCAAGGGCTCCTACGAGAGTG-3’ | 5’-TCGTACTTCTCCTTCAGGGCATCAC-3’ |

Forward and reverse primers used in quantitative PCR were listed.

**Supplementary Figure Legends**

**Figure S1. GREB1 Is4 is expressed in melanoma**

A. *GREB1* mRNA expression in various cancer cell lines from the GENT2 dataset are presented. The data distribution across the dataset is visualized with box plots, as shown in Fig. 1A.

B. Lysates from 293T cells expressing FLAG-tagged full-length, N-terminal half, C-terminal half (Is4) GREB1, and fragments A, B, C, ΔA, ΔB, and ΔC GREB1 Is4 mutants were probed with anti-FLAG antibody and different anti-GREB1 antibodies (#1 and #2). Schematic representations depict the GREB1 and GREB1 Is4 mutant protein structures. NLS indicates nuclear localization sequence.

C. Widmer plot of Melanocytic (Proliferative (R)) (x-axis) versus Mesenchymal-like (Invasive (R)) (y-axis) correlation coefficients of melanoma cell lines used in Fig. 1C. The *R* value was derived from HOPP data (http://jurmo.ch/hopp/hopp_default_data.php).

D. Schematic representations of qPCR primers for *GREB1 gene*. The *GREB1 Is4* gene encodes amino acids 1003 to 1949 of full-length GREB1 but lacks the NLS. GREB1 cDNA corresponding to N-ter1 (270–365 aa), N-ter2 (684–766 aa), C-ter1 (1362–1442 aa), and C-ter2 (1718–1772aa) were amplified in Fig. 1D.

E. The transcription levels for each GREB1 exon in representative cancer types from TCGA dataset are shown. Exon RNA-seq data were derived from TSVdb (http://www.tsvdb.com/index.html). Cancer types with very low transcript levels of the whole *GREB1* locus are not listed. A schematic representation of the human *GREB1* locus is shown below the graph. The closed triangles indicate the location of exon 1 and 19. Exon numbers were assigned based on the GREB1 transcription ID: ENST00000381486.7 (GREB1-204).

F. *GREB1* transcribed regions in normal skin, cervix and ovary tissues from GTEx datasets are shown. A schematic representation of the human *GREB1* locus is shown below the graph. The closed triangles indicate the location of exon 1 and 19.

**Figure S2. MITF stimulates *GREB1 Is4* gene expression**

A. Scatter plots between *GREB1* (x-axis) versus ER signaling gene (ESR1) (y-axis) from the Breast-BRCA TCGA dataset (N = 1097) or between the *GREB1* (x-axis) and *AXIN2* gene (y-axis) from the Lopez hepatoblastoma dataset (N = 55). The solid line indicates linear fit. *R* and *P* indicate Pearson’s correlation coefficient and *P*-value, respectively.

B. Scatter plots for *GREB1* (x-axis) versus *MITF* (y-axis) expression from the DepMap gene expression 21Q4 public database of cancer cell lines (N = 1389). The solid line was drawn using only the skin cancer cell line data (N = 85) and indicates a linear fit. *R* and *P* indicate Pearson’s correlation coefficient and *P*-value, respectively, among the skin cancer cell lines.

C. SKMEL28 and G361 cells were treated with control or MITF siRNA. The mRNA expression of the *MITF*, *GREB1 Is4*, and *MITF* target gene (*MLANA*) were measured by qPCR. The results are shown as fold-changes compared with those of cells treated with control siRNA, and expressed as mean ± s.d. of two independent experiments. *, *P* < 0.05; **, *P* < 0.01, Student's t-test. Bottom panels, lysates from SKMEL28 and G361 cells treated with control or MITF siRNA were probed with an anti-GREB1(#2), anti-MITF or anti-HSP90 antibody.

D. Colo679 cells expressing control vector or exogenous MITF were treated with control or MITF siRNA. The mRNA expression of the *MITF*, *GREB1 Is4*, and *MITF* target genes (*MLANA*, *DCT* and *PMEL*) were measured by qPCR. The results are shown as fold-changes compared to those of cells expressing control vector and treated with control siRNA, and expressed as mean ± s.d. of three independent experiments. **, *P* < 0.01; ***, *P* < 0.001, Student's t-test.

E. 293T cells were cotransfected with FLAG-HA-MITF, a reporter construct containing either GREB1 Is4 promoter (-82-0) or (-315-0) and β-galactosidase gene. The luciferase activities were normalized by β-galactosidase. The data are shown as the fold-change relative to the pGL4 vector control.

F. Lysates from 293T cells expressing control vector and FLAG-HA-MITF with reporter constructs in Fig. 2E were probed with an anti-FLAG, anti-Luciferase, or anti-HSP90 antibodies. Non-specific bands are marked with an asterisk.

**Figure S3. Single cell analysis of *GREB1 gene* in melanoma**

A-C. tSNE plots of single-cell transcriptomic profiles from nine patient-derived cultures and A375 cell line are shown. Clusters were annotated as four states (A). tSNE plots of GREB1 (B) and MITF (C) are shown. The area of A375 or melanocytic cells is surrounded by a line.

D-F. UMAP plots of single-cell transcriptomic profiles from PDX melanoma cells isolated at different time points of treatment are shown. UMAP plot of NCSC gene signature (D), GREB1 (E) and MITF (F) are shown. The area of NCSC gene signature-positive cells is surrounded by a red line.

**Figure S4. Validation of anti-GREB1 and anti-MITF antibodies for IHC**

A. Melanoma tissue was stained only with anti-mouse IgG alkaline phosphatase (AP) (secondary) (a) or with anti-GREB1 (#2) (primary) and secondary antibodies (b). Another melanoma tissue was stained only with anti-rabbit IgG AP (secondary) antibody (c) or with anti-MITF (primary) and secondary antibodies (d). The sections were stained with Warp Red Chromogen Kit and hematoxylin as shown in Figs. 3A and B. Non-specific staining was tested by staining with secondary antibody only. Scale bars, 100 μm.

B. Colo679 melanoma cells treated with control or GREB1 (#1) or MITF siRNA were stained with anti-GREB1 (#2) (a, b) or anti-MITF (c, d) antibody. Scale bars, 400 μm.

C. Representative photomicrographs of GREB1 and MITF IHC staining in melanoma tissues. The sections were stained using the Warp Red Chromogen Kit and counterstained with hematoxylin, as shown in Figs. 3A and B. GREB1 and MITF expression levels in the tumors were scored as 0 (a, e), 1 (b, f), 2 (c, g), and 3 (d, h). Scale bars, 100 μm.

**Figure S5. GREB1 and MITF expression is associated with prognosis of melanoma cases**

A. GREB1 IHC scores for melanoma specimens (N = 89) (left panel) and *GREB1* mRNA expression from the Skin-TCGA dataset (N = 470) (right panel) were plotted by pathological clinical stage. The normal distribution across melanoma patients is illustrated with box and dot plots, as described in Fig. 1A.

B, C. The relationships between overall survival and GREB1 or MITF expression levels or tumor thickness in melanoma patients were analyzed. The data were obtained from our melanoma specimens (n = 89) (B) or the Skin-TCGA melanoma dataset (N = 474) (C). The log-rank test was used for statistical analysis.

**Figure S6. GREB1 Is4 is required for melanoma cell proliferation**

A. Lysates from Colo679 and SKMEL28 cells treated with control, GREB1 #1, or GREB1 #2 siRNA were probed with anti-GREB1 (#2) or anti-HSP90 antibody. HSP90 was used as a loading control.

B. A375 (left panel) and MM-RU (right panel) cells were transfected with 10 nM control or two GREB1 siRNAs and then subjected to the 2D cell proliferation assay using CyQUANT NF. Fluorescence intensity was measured at the indicated time points. The data are presented as the mean ± s.d. for three independent experiments.

C. Left panel, Colo679 cells were treated with 10 nM control or two GREB1 ASOs and cultured for the indicated number of days. Cell numbers were quantified using CyQUANT NF. Right panel, total RNA was isolated from the cells used in the 2D cell proliferation assay. *GREB1* mRNA levels were quantified by qPCR. The data were normalized to *GAPDH* and expressed as the fold-change in *GREB1* mRNA levels versus the cells treated with control ASO.

D. Lysates from Colo679 cells expressing GFP or GREB1 Is4 were probed with anti-GREB1 and anti-HSP90 antibodies. This figure shows GREB1 Is4 expression in the cells used in Fig. 4D.

E. Lysates from Dox-inducible A375/GREB1 Is4 cells treated with various concentrations of Dox for 2.5 or 5 hr were probed with anti-GREB1 (#2) and anti-HSP90 antibodies. In Fig. 4E, A375/GREB1 Is4 cells were treated with 20 ng/ml Dox.

F. Colo679 cells expressing control vector or GREB1 Is4 were transfected with control or two GREB1 siRNAs. The lysates were probed with anti-GREB1 (#2) or anti-HSP90 antibody. HSP90 was used as a loading control.

G. MeWo cells expressing control vector or GREB1 Is4 were transfected with control or two GREB1 siRNAs and subjected to the 2D cell proliferation assay. The data are presented as the mean ± s.d. from three independent experiments (*, *P* < 0.05 and **, *P* < 0.01, Student's t-test).

H. MeWo cells expressing control vector or GREB1 Is4 were transfected with control or two GREB1 siRNAs. The lysates were probed with anti-GREB1 (#2) and anti-clathrin antibodies. Clathrin was used as a loading control.

**Figure S7. Reduced GREB1 Is4 affects gene expression in melanoma cells**

A. The equal amounts (15 μg of proteins) of total lysates from parental, vector control or GREB1 Is4 KO Colo679 clones were probed with anti-GREB1 #2, anti-MITF and anti-HSP90 antibodies. HSP90 was used as a loading control.

B. Left panel, GREB1 Is4 or MITF KO Colo679 cell lines were cloned. Three clones each of empty vector-transfected control, GREB1 Is4 KO, and MITF KO cells were cultured for the indicated days, and the cell numbers were quantified using CyQUANT NF. Data are presented as the fold-change in cell number relative to day 0. The results are presented as the mean ± s.d. from three independent experiments. Right panel, the average cell numbers on day 7 for the vector control (N = 6), GREB1 Is4 KO (N = 10), and MITF KO (N = 4) clones are plotted. Bars show the median of each cell line clone (*, *P* < 0.05 and ***, *P* < 0.001 by the Student’s t-test).

C. Left panel, scatter plots for the relationship between the *GREB1* Gene Effect (DEMETER2) RNAi (x-axis) and *GREB1* expression 21Q4 Public (y-axis) across 488 cell lines in the Depmap dataset. Right panel, scatter plots for the relationship between the *MITF* Gene Effect (Chronos) CRISPR (x-axis) and *MITF* expression 21Q4 Public (y-axis) across 973 cell lines in the Depmap dataset. Colo679 and A375 cells are indicated in red and orange, respectively.

D. Colo679 cells expressing control vector or GREB1 Is4 were transfected with control or GREB1 siRNA. The mRNA expression of the proliferative (*GREB1 Is4, CDK2, PLK1, MITF or MCM*5) and CDKi (*CDKN1A*) genes were measured by qPCR. The results are shown as fold-changes compared with those of cells expressing control vector and treated with control siRNA, and expressed as mean ± s.d. of two independent experiments.

**Figure S8. GREB1 Is4 mediates melanoma development *in vivo***

A. Diagram of the *WT ROSA 26* targeting construct and the *GREB1 Is4 Tg* allele.

B. Insertion of the targeting construct into ES cells was confirmed by PCR for the left and right arm regions of the GREB1 Is4 allele. The PCR primer sets are depicted in A. The *rosa 26* region was used as a control.

C. Lysates from tumors resected from *Is4^-/-^* (control) and *Is4^+/-^* (GREB1 Is4) in *Tyr-CreER^+/-^; BRAF^V600E/WT^; PTEN^flox/WT^* melanoma mice were probed with the indicated antibodies. GREB1 Is4 is tagged with FLAG-HA. HSP90 was used as a loading control.

D. Tumor specimens from control (a) and GREB1 Is4 (b) melanoma mice were stained with an anti-HA antibody to detect FLAG-HA-GREB1 Is4. Scale bars, 100 μm.

E Mouse *Greb1* and *Pmel* mRNA expression in NIH3T3, positive control cell line, *BRAF^V600E/WT^*; *PTEN^lox/WT^* melanoma tissue, BL16BL6, B16F10 or Yummner1.7 mouse melanoma cell lines were measured by qPCR. Ct indicates the threshold cycle number of qPCR.

F Schematic representations of mouse Greb1 and human GREB1 isoform structures from ensemble data (https://asia.ensembl.org/index.html). Only protein-coding splicing variant isoforms were selected. The gray areas indicate amino acid sequences that are different from the full-length GREB1 (GREB1-201), and GREB1-206 corresponding to Is4 is shown in red.

**Figure S9. Relationship between GREB1 Is4 and CAD**

A. Lysates from HeLa and U2OS cells expressing GFP (control) or FLAG-HA-GREB1 Is4 were immunoprecipitated with an anti-FLAG antibody. The immunoprecipitates were eluted with FLAG peptide, and the FLAG-HA-GREB1 Is4-binding proteins were detected using silver staining and analyzed by LC-MS/MS. FLAG-HA-GREB1 Is4 (bait) is indicated by a red arrowhead. The other identified bands are listed in Supplementary Tables S4 and S5.

B. Colo679 cells expressing FLAG-HA-GREB1 Is4 or MYC-CAD were stained with the anti-FLAG or anti-MYC antibodies, and counterstained with Phalloidin and Hoechst33342. Phalloidin or Hoechst33342 was used as a F-actin or nucleus staining marker, respectively. Scale bar, 10 μm.

C. Lysates from 293T cells expressing the MYC-CAD domains (GLNase, CPSase or DHO and ATCase) and FLAG-HA-GREB1 Is4 were immunoprecipitated with an anti-FLAG antibody, and the immunoprecipitates were probed with anti-MYC or anti-FLAG antibody. A schematic representation of the structure of human CAD is shown below. Non-specific bands are marked with asterisks.

**Figure S10. GREB1 Is4 is required for pyrimidine synthesis.**

A. The schematic diagram depicts the *de novo* pyrimidine synthesis pathway and the pyrimidine base ring incorporating the nitrogen from glutamine and aspartic acid, and carbon atoms from CO_2_.

B. Colo679 cells transfected with control or GREB1 #1 siRNA were labeled with ^15^N_2_^13^C_5_-glutamine for 60 min, and the intracellular concentrations of the indicated metabolites were measured using LC-QEMS analysis. When CAP becomes CAA ^15^N of aspartic acid (^15^N is supplied to Asp by the Gln->Glu->Asp pathway) is added, metabolites of the pyrimidine pathway also have two ^15^N (M+2) except for CAP. As in Fig. 7B, the amounts of metabolites(M+2) were plotted. Experiments were performed in quadruplicate. The data are presented as the mean ± s.d. (**, *P* < 0.01; ***, *P* < 0.001, Student’s *t*-test).

C. The enrichment of labeled (M+1 + M+2) to total metabolite (M+0 + M+1 + M+2) is shown. This ratio was calculated using the data obtained from Fig. 7B and Supplementary Fig. S10B. The data are presented as the mean ± s.d. (**, *P* < 0.01; ***, *P* < 0.001, Student’s *t*-test).

D. Colo679 transfected with control or two GREB1 siRNAs were treated with 50 μM CTU (cytidine, thymidine and uridine nucleosides) and subjected to the 2D cell proliferation assay using CyQUANT NF. The data are presented as the mean ± s.d. from three independent experiments.

**Figure S11. Sleeping Beauty (SB) transposon insertion sites in the *Greb1* locus from SBCDDB (Sleeping Beauty Cancer Driver Database)**

SB transposon insertion sites in the *Greb1* locus were derived from SBCDDB web site (https://sbcddb.moffitt.org). The insertion sites in BRAF melanoma mice (MEL) are indicated by red arrows. In the forward orientation (right-pointing black arrowheads), the transposon can drive expression of downstream exons using the internal promoter and splice donor. In the reverse orientation (left-pointing black arrowheads), the transposon terminates expression of exons downstream of the insertion site. The closed blue triangle indicates the location of exon 1 and 19. Exon numbers were assigned based on the *greb1* transcription ID: ENSMUST00000048064.16 (Greb1-201). The locus corresponding to human GREB1 Is4 is indicated by a black double-headed arrow.

**Figure S12. GREB1 expression is associated with prognosis, *BRAF* mutation, and CD8A cell infiltration in melanoma cases.**

A. Left panel, in the melanoma SKCM TCGA dataset, *BRAF* mutations were mapped at the *BRAF* locus, and *GREB1* mRNA expression is shown as a heat map. The maps were created using the UCSC Xena website. Right panel, *GREB1* mRNA levels in the melanoma SKCM TCGA dataset with WT *BRAF* or mutated (Mut) *BRAF* are plotted. The data distribution across the dataset is visualized with box plots.

B. The relationship between overall survival and *GREB1* expression in the melanoma SKCM TCGA dataset with WT *BRAF* or Mut *BRAF* is plotted. The log-rank test was used for statistical analysis.

C. The relationship between overall survival and *GREB1* expression in the melanoma SKCM TCGA dataset with low or high *CD8A* expression is plotted. The log-rank test was used for statistical analysis.
