# Supplementary figures and images for: GREB1 isoform 4 is specifically transcribed by MITF and required for melanoma proliferation
Source: Oncogene. 2023 Sep 1;42(42):3142–56. doi: 10.1038/s41388-023-02803-6 (PMC10575781; doi:10.1038/s41388-023-02803-6)

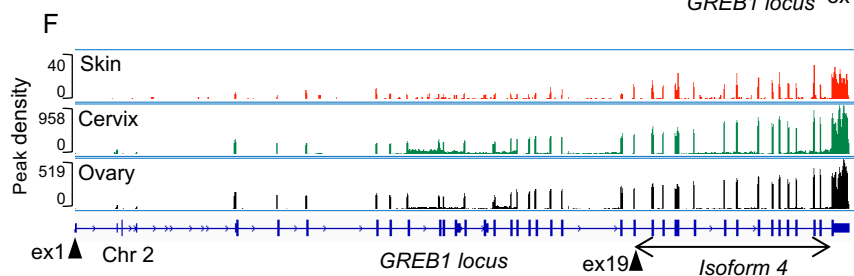

Supplement: Supplementary file 2 — Supplementary Figure1 [file 41388_2023_2803_MOESM2_ESM.pdf]

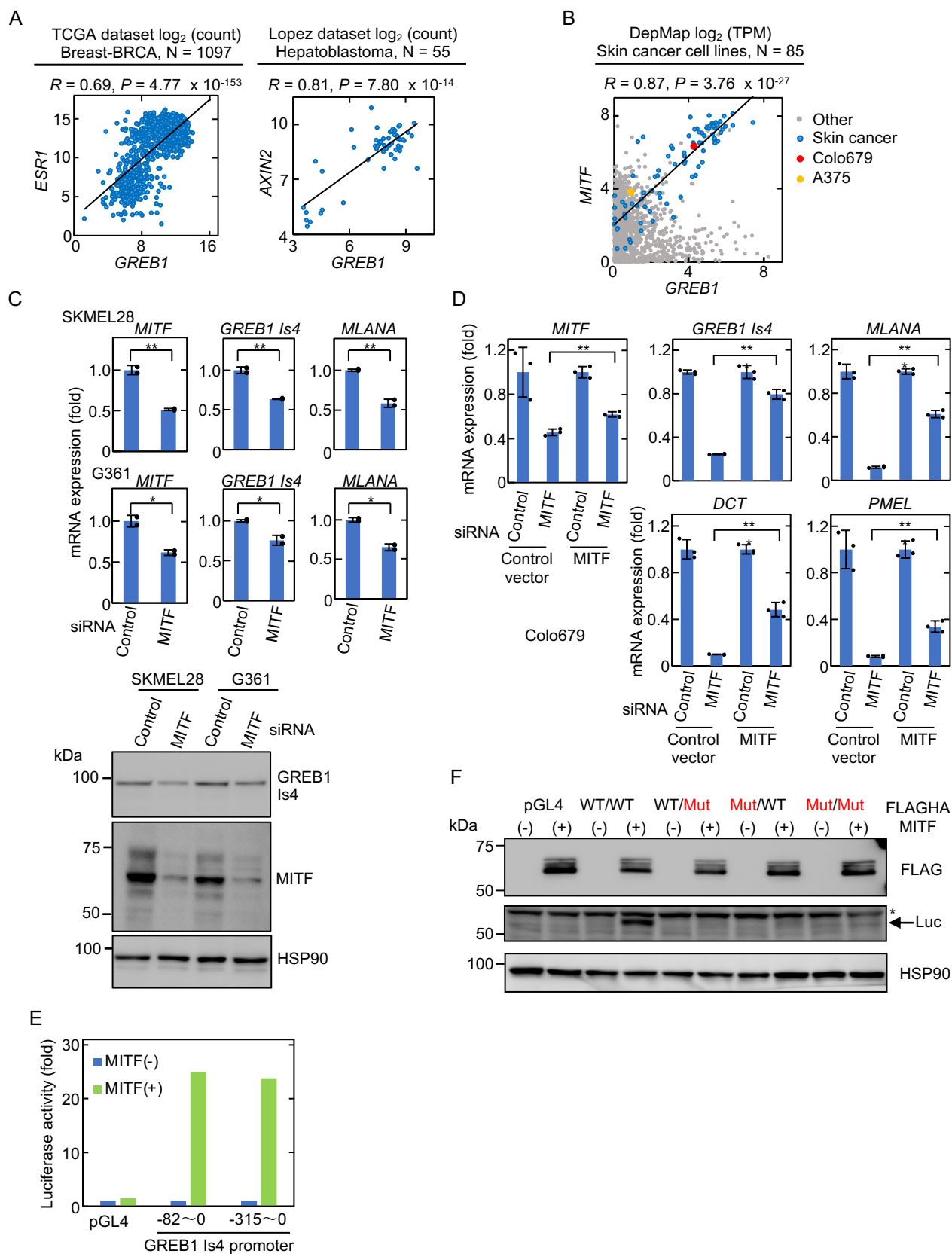

Supplement: Supplementary file 3 — Supplementary Figure2 [file 41388_2023_2803_MOESM3_ESM.pdf]

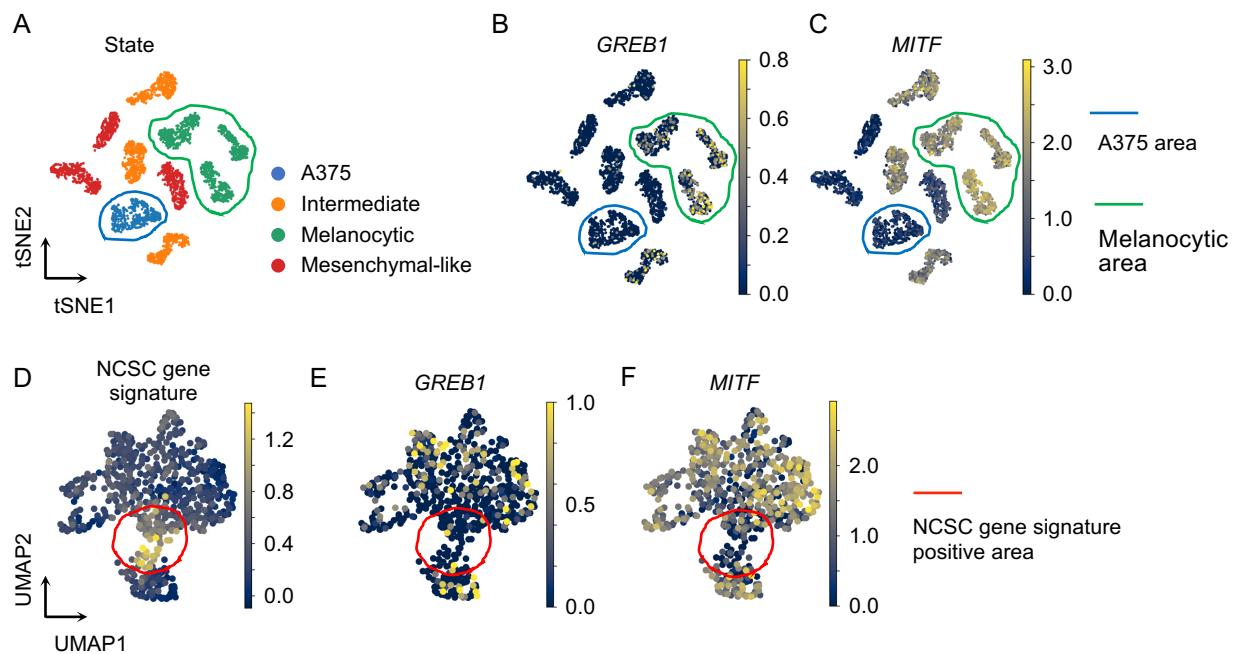

Supplement: Supplementary file 4 — Supplementary Figure3 [file 41388_2023_2803_MOESM4_ESM.pdf]

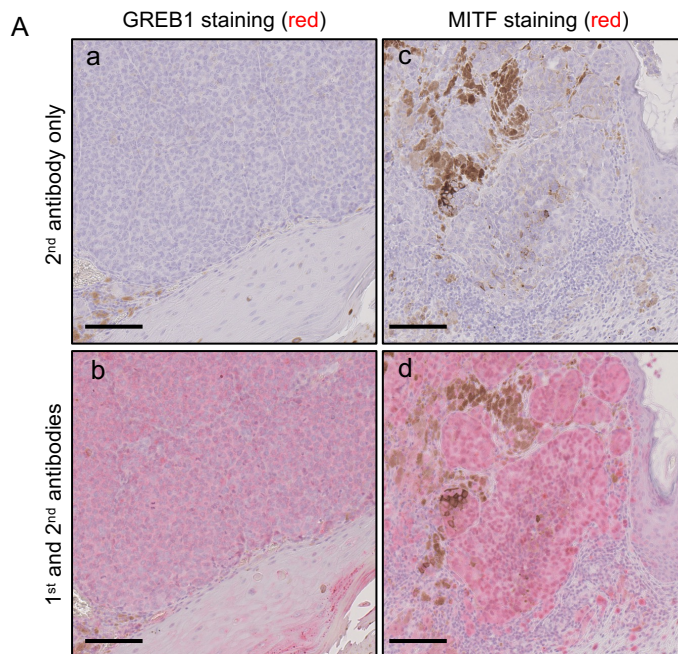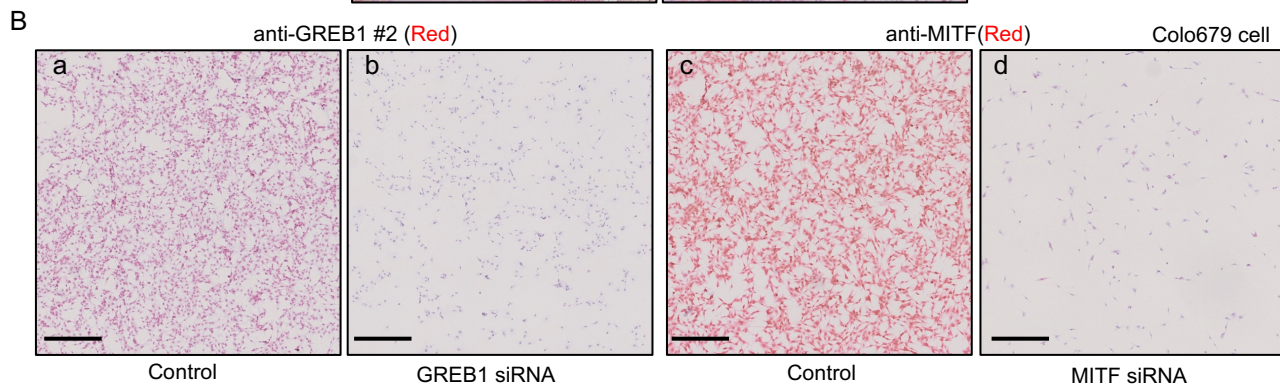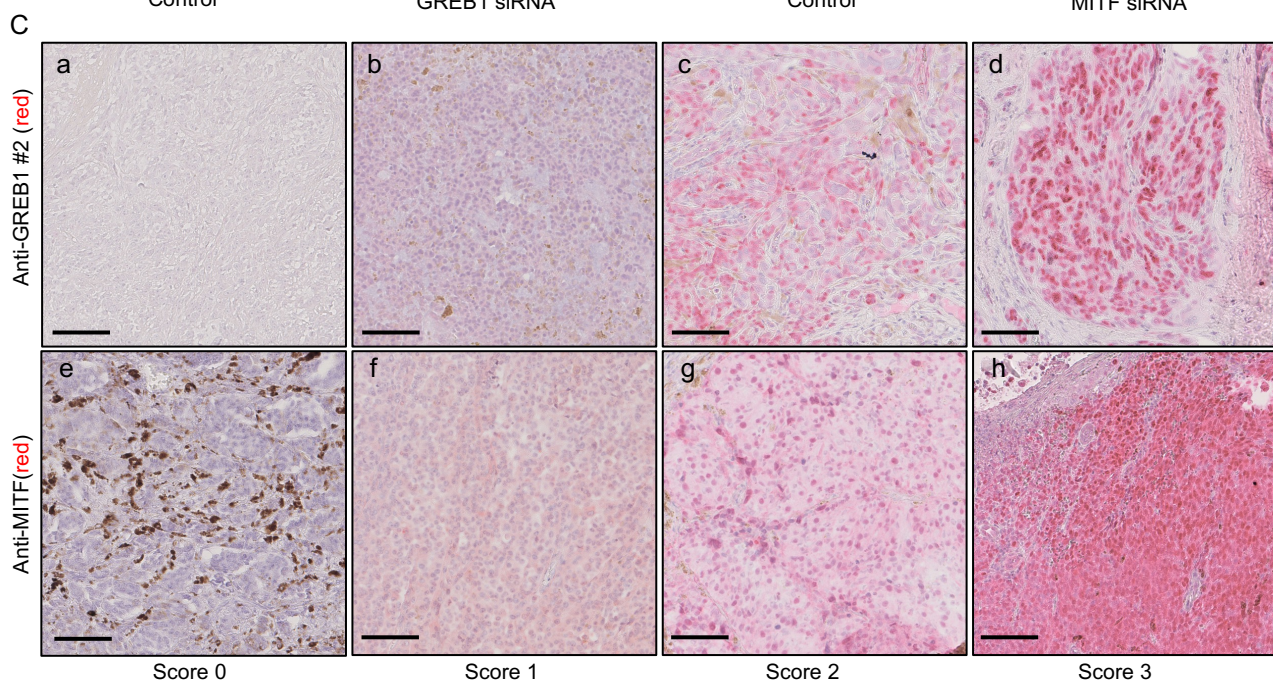

Supplement: Supplementary file 5 — Supplementary Figure4 [file 41388_2023_2803_MOESM5_ESM.pdf]

A

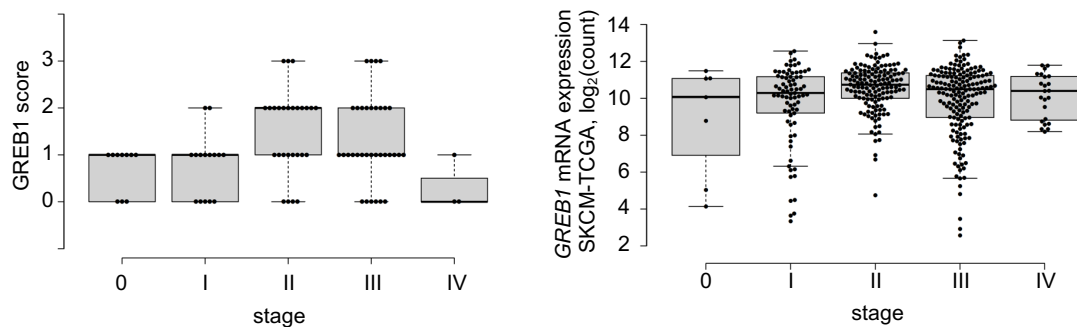

B

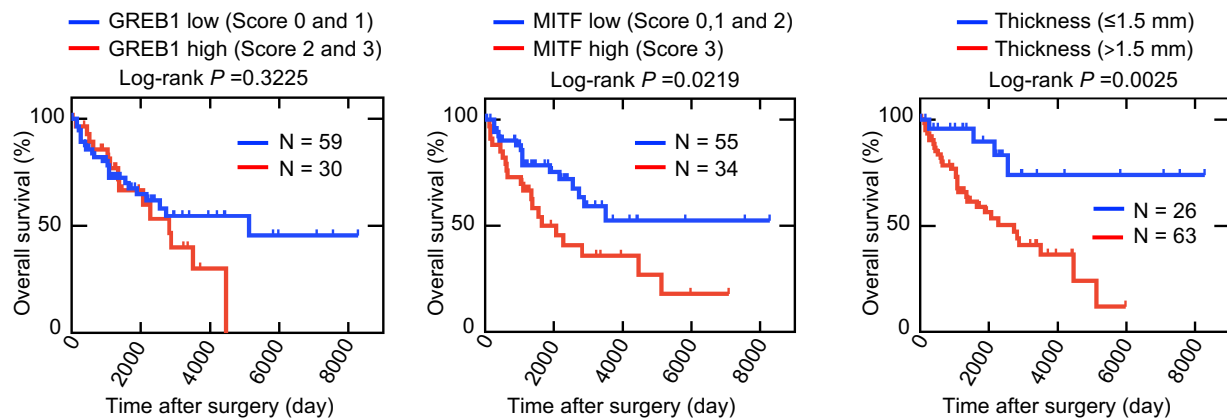

C

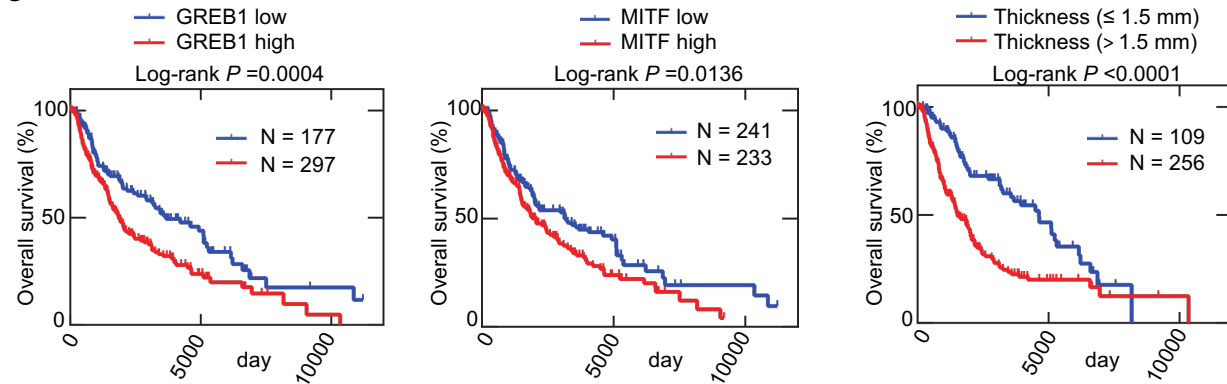

Supplement: Supplementary file 6 — Supplementary Figure5 [file 41388_2023_2803_MOESM6_ESM.pdf]

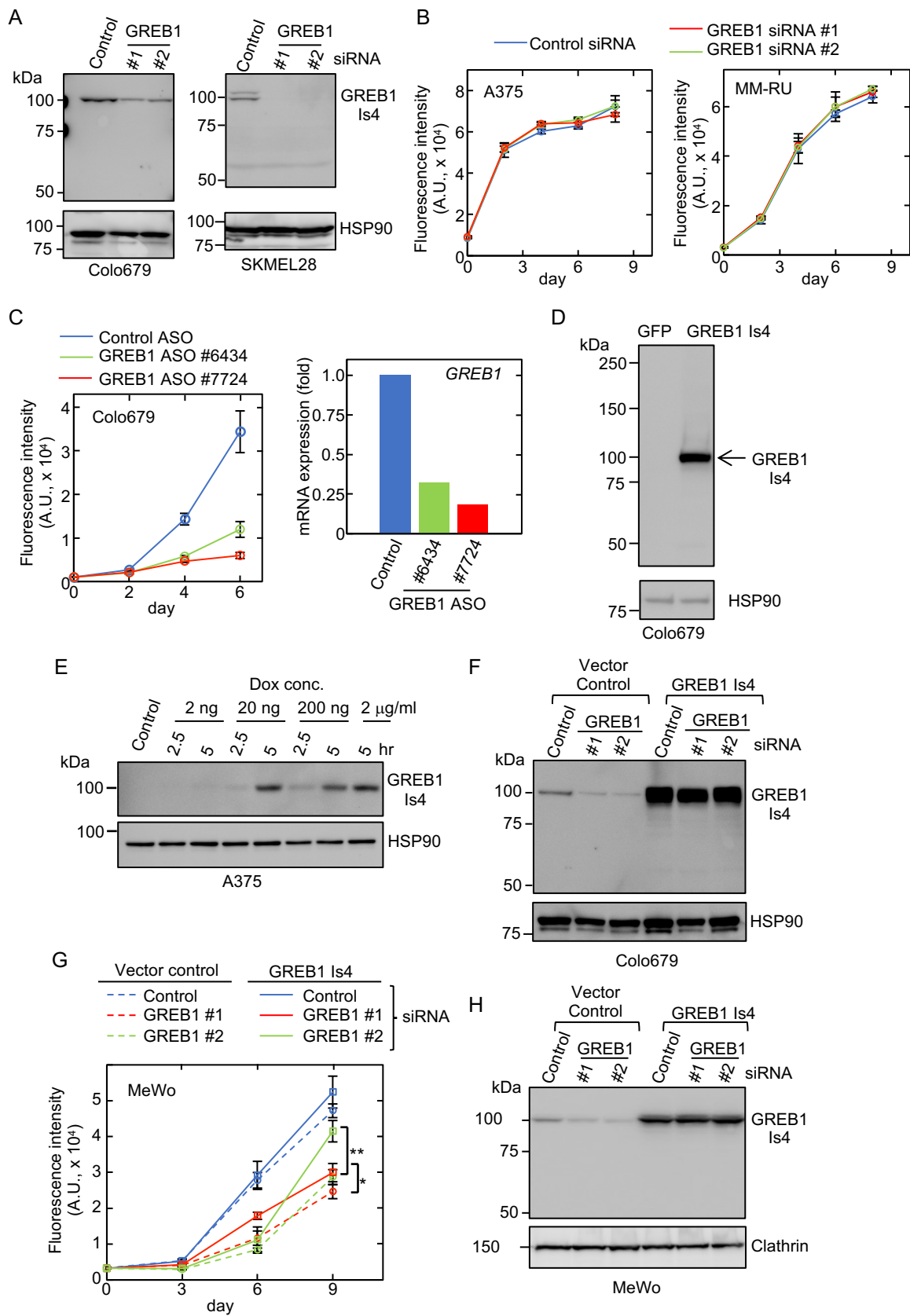

Supplement: Supplementary file 7 — Supplementary Figure6 [file 41388_2023_2803_MOESM7_ESM.pdf]

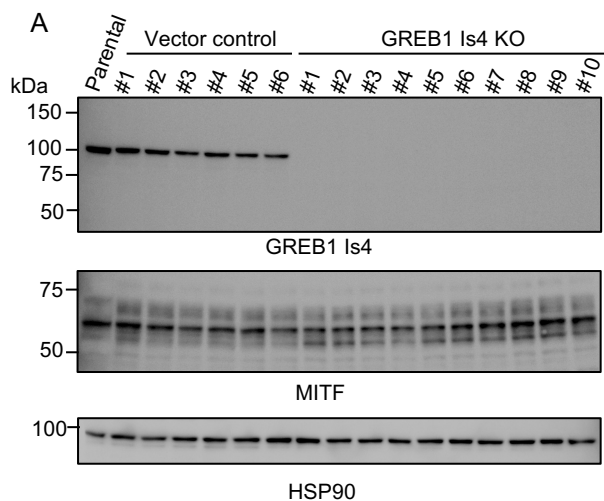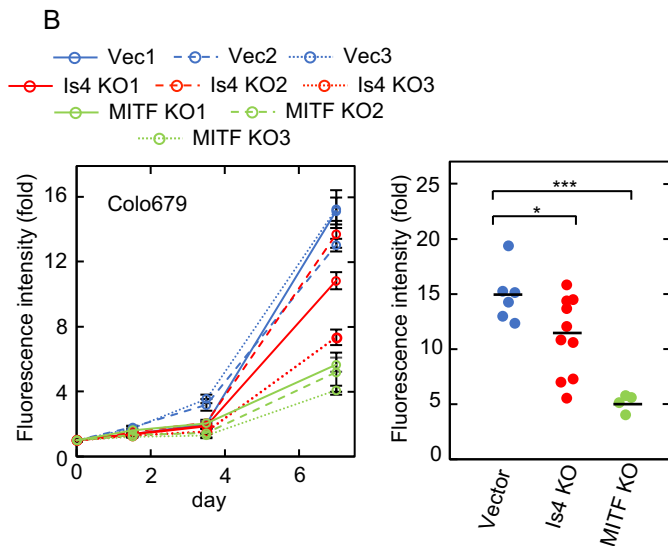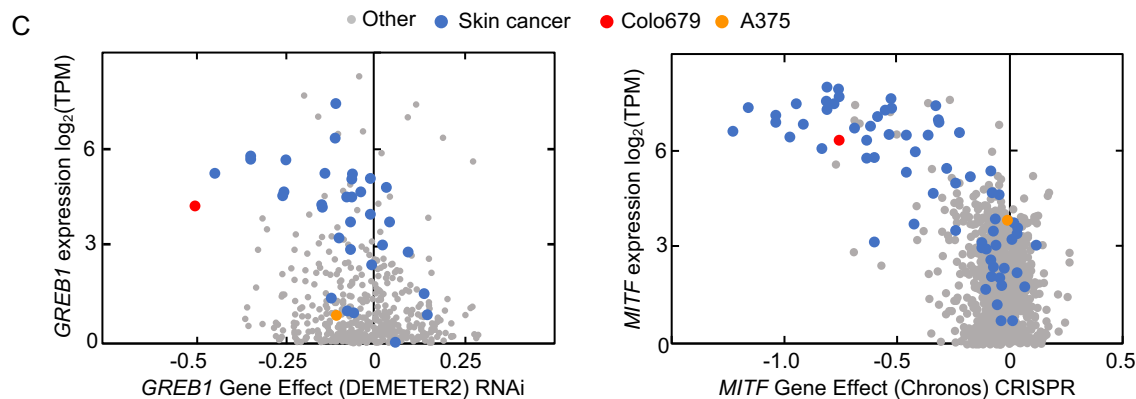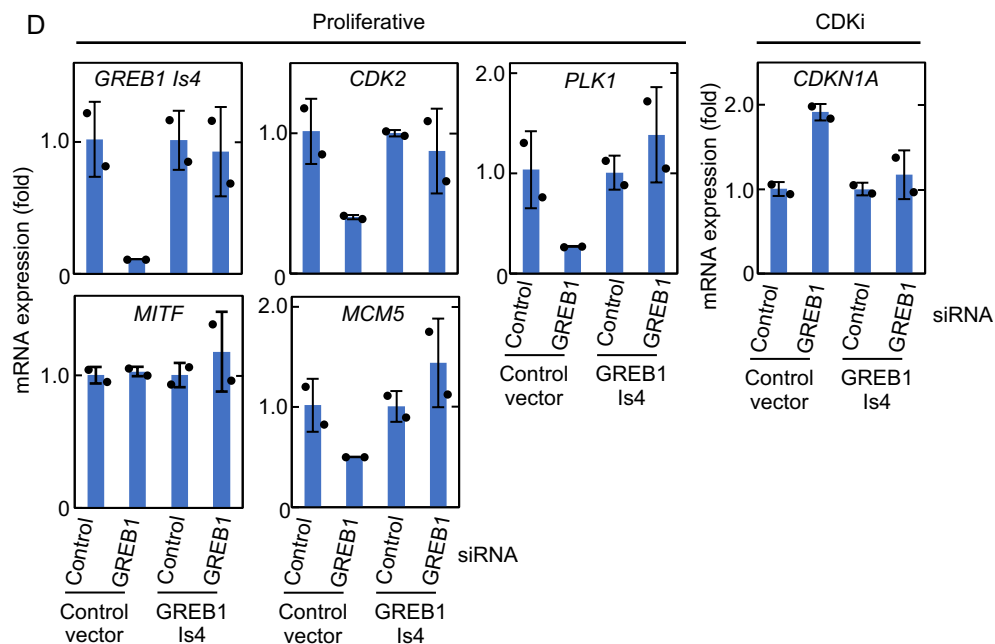

Supplement: Supplementary file 8 — Supplementary Figure7 [file 41388_2023_2803_MOESM8_ESM.pdf]

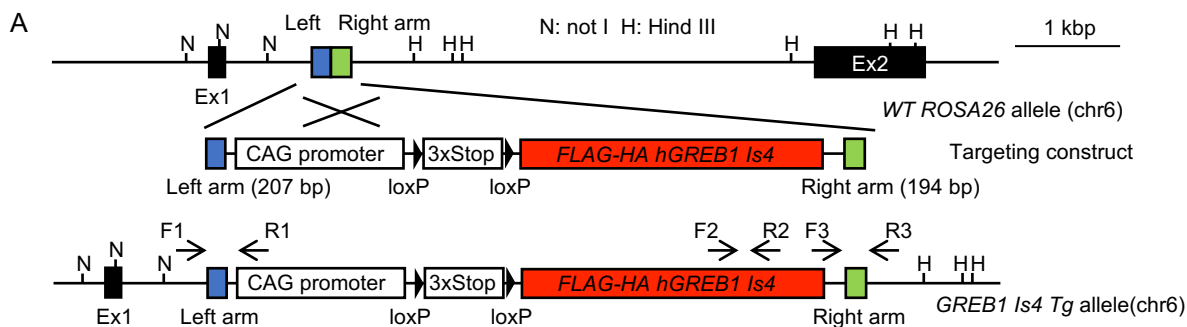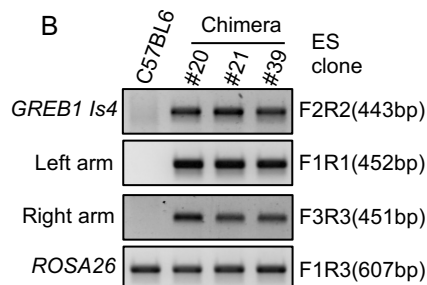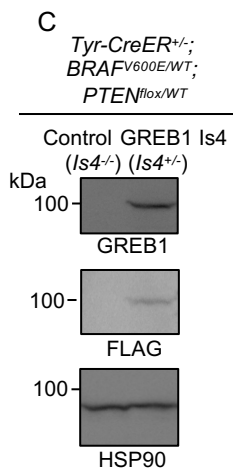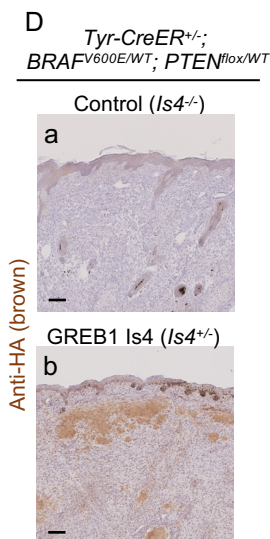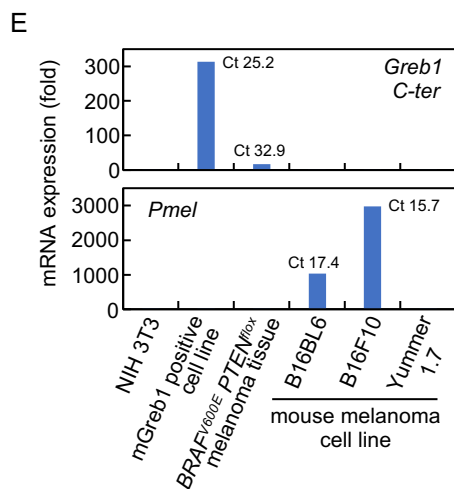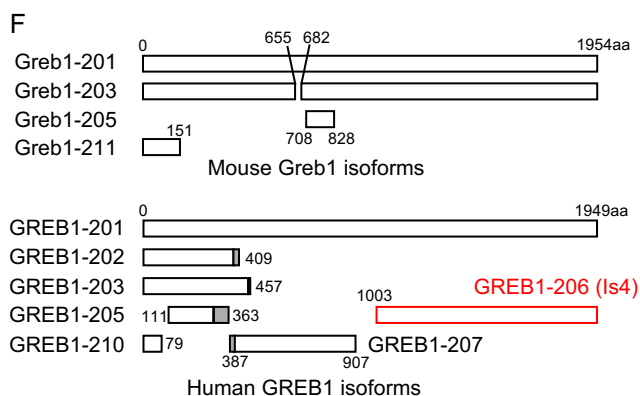

Supplement: Supplementary file 9 — Supplementary Figure8 [file 41388_2023_2803_MOESM9_ESM.pdf]

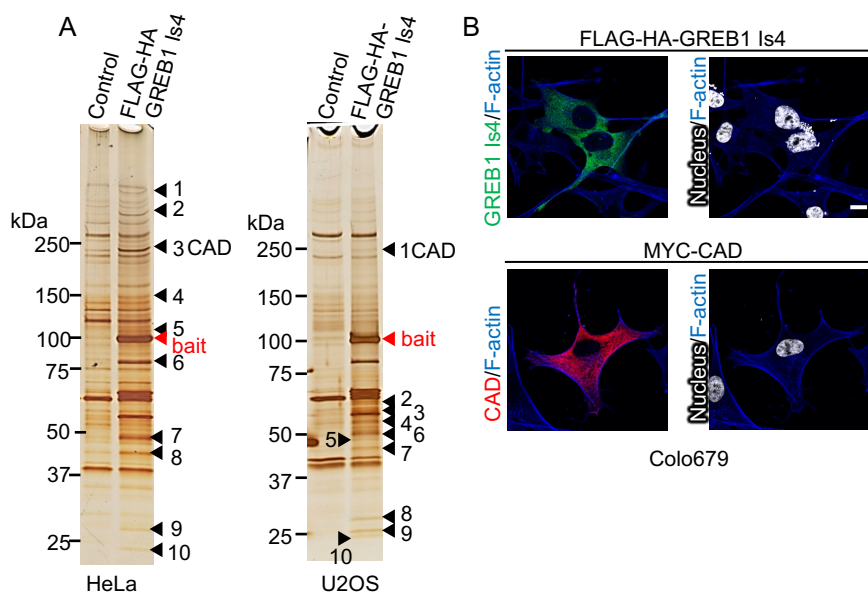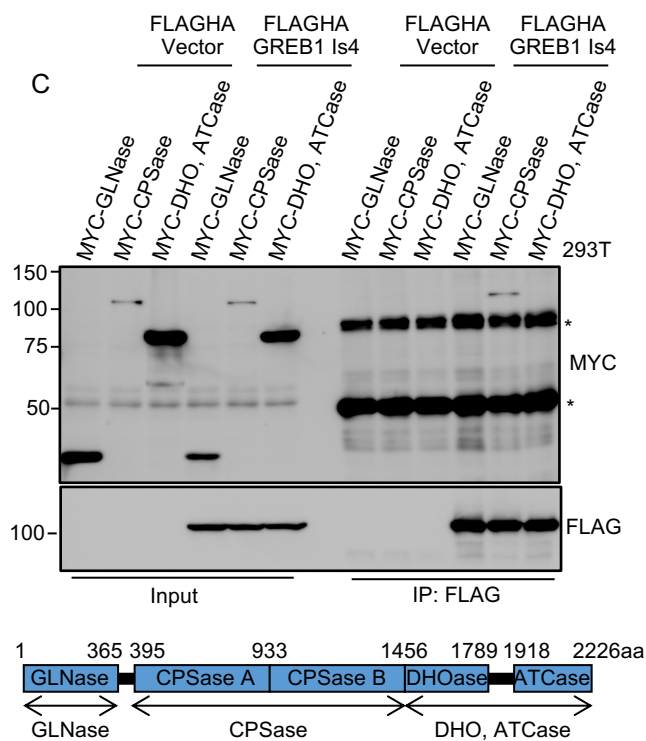

Supplement: Supplementary file 10 — Supplementary Figure9 [file 41388_2023_2803_MOESM10_ESM.pdf]

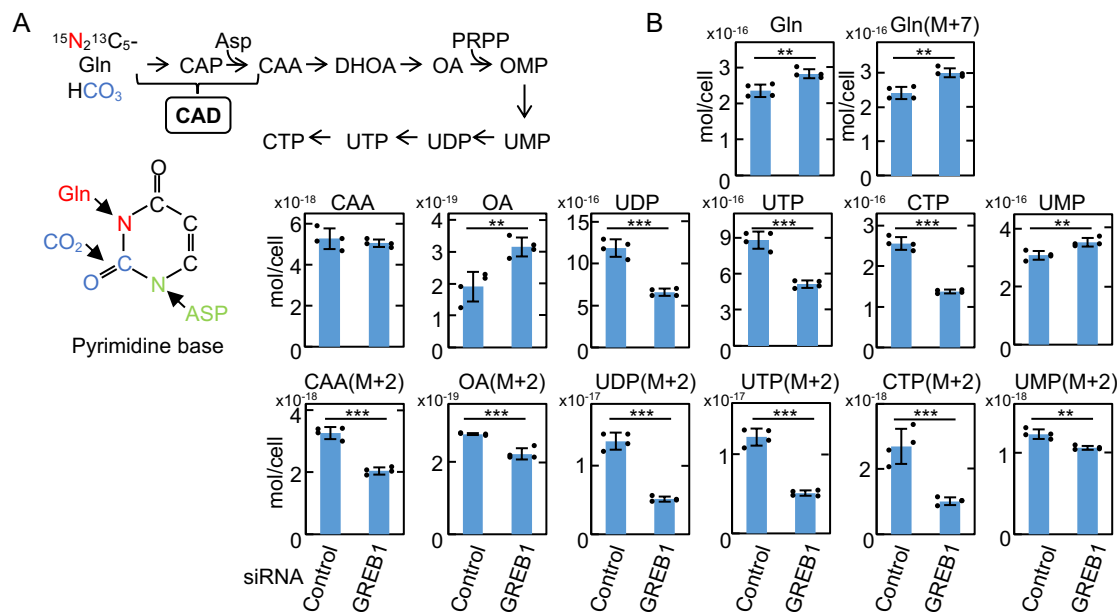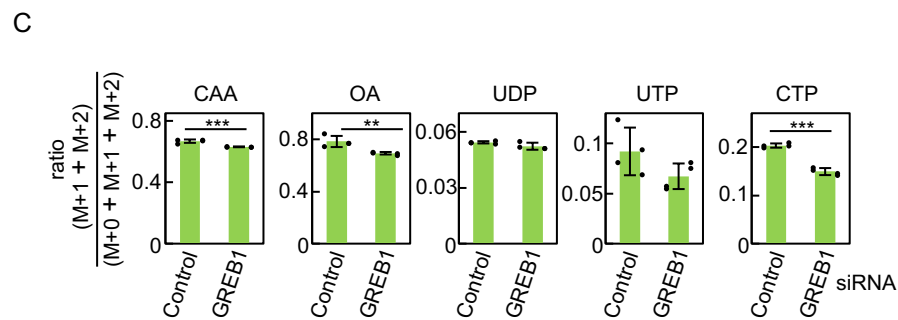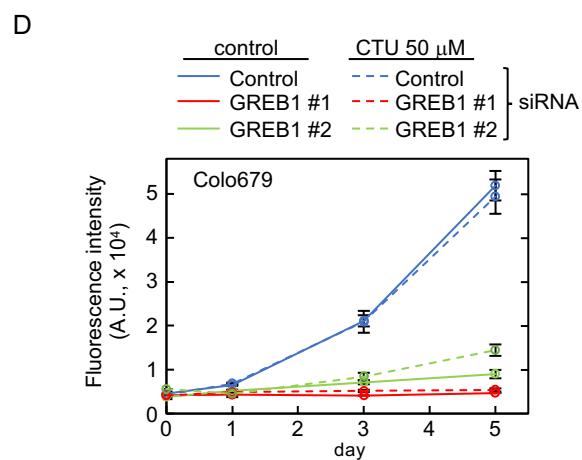

Supplement: Supplementary file 11 — Supplementary Figure10 [file 41388_2023_2803_MOESM11_ESM.pdf]

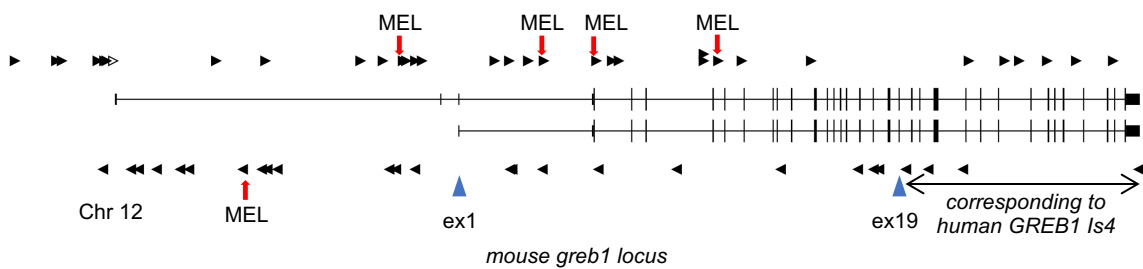

Supplement: Supplementary file 12 — Supplementary Figure11 [file 41388_2023_2803_MOESM12_ESM.pdf]

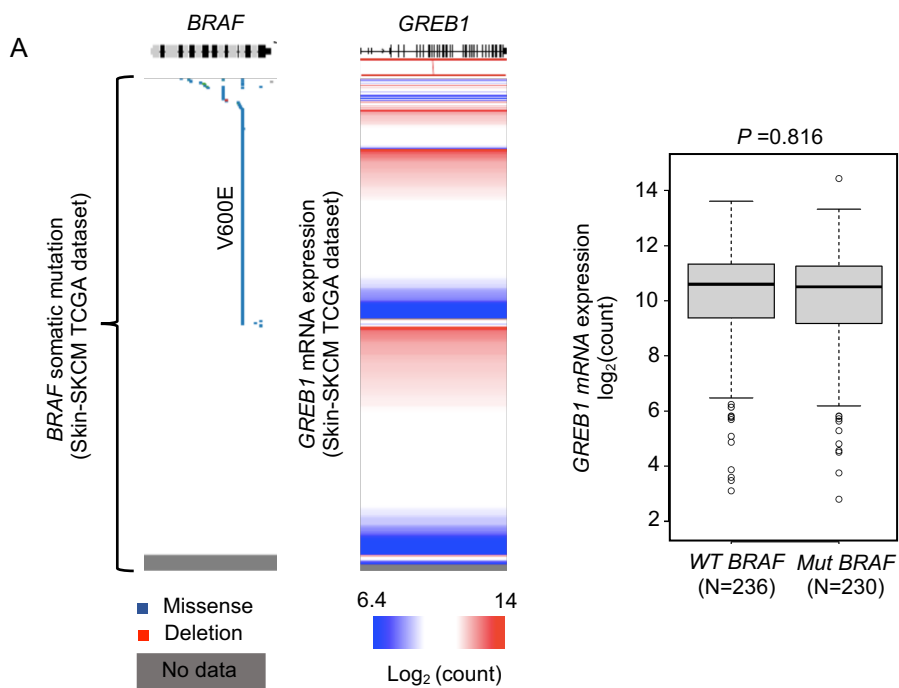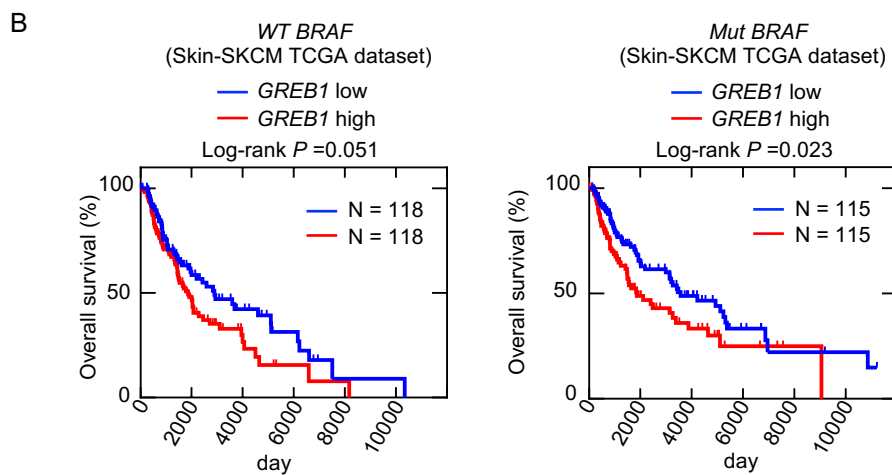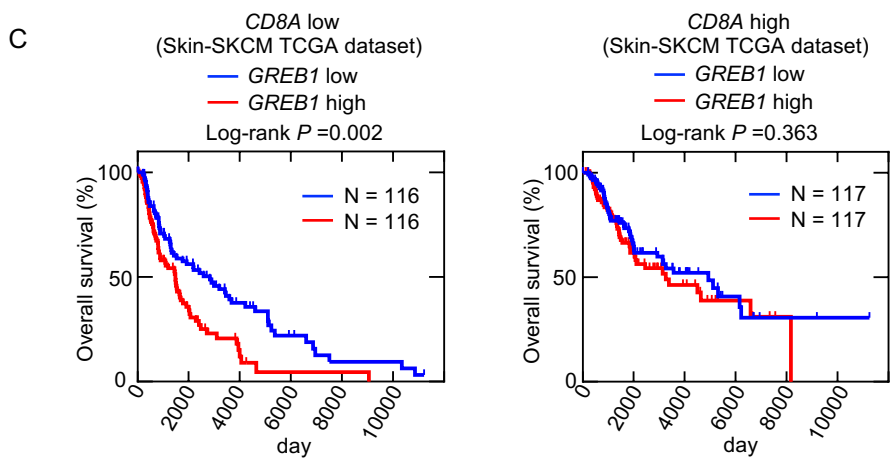

Supplement: Supplementary file 13 — Supplementary Figure12 [file 41388_2023_2803_MOESM13_ESM.pdf]
